# Multi-scale and multi-parametric radiomics of gadoxetate disodium–enhanced MRI predicts microvascular invasion and outcome in patients with solitary hepatocellular carcinoma ≤ 5 cm

**DOI:** 10.1007/s00330-020-07601-2

**Published:** 2021-01-14

**Authors:** Huan-Huan Chong, Li Yang, Ruo-Fan Sheng, Yang-Li Yu, Di-Jia Wu, Sheng-Xiang Rao, Chun Yang, Meng-Su Zeng

**Affiliations:** 1 Shanghai Institute of Medical Imaging, 180 Fenglin Road, Shanghai, China; 2 Department of Radiology, Zhongshan Hospital, Fudan University, 180 Fenglin Road, Shanghai, 200032 China; 3 Shanghai United Imaging Intelligence Co., Ltd, Shanghai, China; 4 Department of Medical Imaging, Shanghai Medical College, Fudan University, Shanghai, China

**Keywords:** Hepatocellular carcinoma, Magnetic resonance imaging, Neoplasm recurrence

## Abstract

**Objectives:**

To develop radiomics-based nomograms for preoperative microvascular invasion (MVI) and recurrence-free survival (RFS) prediction in patients with solitary hepatocellular carcinoma (HCC) ≤ 5 cm.

**Methods:**

Between March 2012 and September 2019, 356 patients with pathologically confirmed solitary HCC ≤ 5 cm who underwent preoperative gadoxetate disodium–enhanced MRI were retrospectively enrolled. MVI was graded as M0, M1, or M2 according to the number and distribution of invaded vessels. Radiomics features were extracted from DWI, arterial, portal venous, and hepatobiliary phase images in regions of the entire tumor, peritumoral area ≤ 10 mm, and randomly selected liver tissue. Multivariate analysis identified the independent predictors for MVI and RFS, with nomogram visualized the ultimately predictive models.

**Results:**

Elevated alpha-fetoprotein, total bilirubin and radiomics values, peritumoral enhancement, and incomplete or absent capsule enhancement were independent risk factors for MVI. The AUCs of MVI nomogram reached 0.920 (95% CI: 0.861–0.979) using random forest and 0.879 (95% CI: 0.820–0.938) using logistic regression analysis in validation cohort (*n* = 106). With the 5-year RFS rate of 68.4%, the median RFS of MVI-positive (M2 and M1) and MVI-negative (M0) patients were 30.5 (11.9 and 40.9) and > 96.9 months (*p* < 0.001), respectively. Age, histologic MVI, alkaline phosphatase, and alanine aminotransferase independently predicted recurrence, yielding AUC of 0.654 (95% CI: 0.538–0.769, *n* = 99) in RFS validation cohort. Instead of histologic MVI, the preoperatively predicted MVI by MVI nomogram using random forest achieved comparable accuracy in MVI stratification and RFS prediction.

**Conclusions:**

Preoperative radiomics-based nomogram using random forest is a potential biomarker of MVI and RFS prediction for solitary HCC ≤ 5 cm.

**Key Points:**

• *The radiomics score was the predominant independent predictor of MVI which was the primary independent risk factor for postoperative recurrence.*

• *The radiomics-based nomogram using either random forest or logistic regression analysis has obtained the best preoperative prediction of MVI in HCC patients so far.*

• *As an excellent substitute for the invasive histologic MVI, the preoperatively predicted MVI by MVI nomogram using random forest (MVI-RF) achieved comparable accuracy in MVI stratification and outcome, reinforcing the radiologic understanding of HCC angioinvasion and progression.*

**Supplementary Information:**

The online version contains supplementary material available at 10.1007/s00330-020-07601-2.

## Introduction

Hepatocellular carcinoma (HCC) is the sixth most prevalent neoplasm and the third leading cause of cancer death [1]. Despite curative therapies, the outcome of HCC patients remains poor, with 5-year recurrence rates reaching 50–70% after hepatectomy and < 35% after liver transplantation [2–6].

Microvascular invasion (MVI), present in 15–57.1% surgical specimens of HCC [7], is a well-established risk factor for postoperative recurrence [8, 9], even for solitary small HCC [10]. To improve the prognosis of MVI-positive patients, a wide resection margin is recommended [11]. Therefore, preoperative diagnosis of MVI is of great importance for treatment strategies.

MVI is defined as the cancer cell nest in vessels lined with endothelium, which is visible only on microscopy [7, 12] and poses a challenge for non-invasive diagnosis. Recently, preoperatively radiologic hallmarks including non-smooth tumor margin, peritumoral enhancement on arterial phase (AP), and peritumoral hypointensity on hepatobiliary phase (HBP) have shown to be conducive to MVI diagnosis but be inferior to radiomics signatures [13]. As a novel and non-invasive tool, radiomics can high-throughput extract quantitative imaging signatures to improve diagnostic or prognostic accuracy [14], which is also applicable to preoperative MVI and outcome prediction. Being related with postoperative recurrence and metastasis, peritumoral area of HCC is rich in highly invasive cells and susceptible to the formation of MVI [12], where it has been neglected in previous radiomics studies [11, 15, 16]. While gadoxetate disodium–enhanced (Gd-EOB-DTPA) MRI offers the identifiability of small or early HCC and the information of tumor heterogeneity and vascularization [17], previous radiomics studies [11, 13] mainly focused on HBP images for predicting MVI. Thus, it is reasonable to investigate whether radiomics signatures extracted from intratumoral and peritumoral regions on multi-parametric images of Gd-EOB-DTPA MRI may allow more effective MVI prediction.

This study aimed to develop and validate nomograms based on multi-scale and multi-parametric radiomics of Gd-EOB-DTPA MRI for the preoperative MVI and outcome prediction in patients with solitary HCC ≤ 5 cm.

## Materials and methods

### Study design and patients

**Fig. 1:**
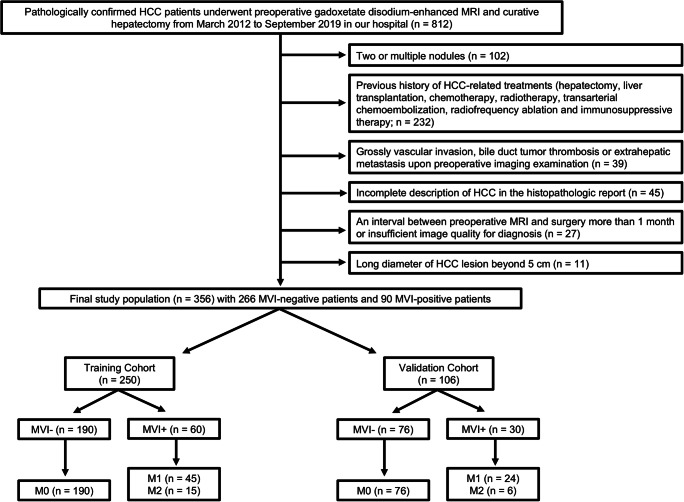
Flowchart of the study population

Our hospital ethics committee approved this retrospective study and waived patient informed consent. Between March 2012 and September 2019, 356 pathologically confirmed HCC patients (303 males and 53 females; 54.22 ± 11.40 years) with preoperative Gd-EOB-DTPA MRI met the inclusion criteria (Fig. 1): (a) solitary HCC with the longest diameter ≤ 5 cm; (b) without gross vascular invasion, bile duct tumor thrombosis or extrahepatic metastasis upon preoperative imaging; (c) without previous history of HCC-related treatments (hepatectomy, liver transplantation, chemotherapy, radiotherapy, transarterial chemoembolization, radiofrequency ablation, and immunosuppressive therapy); (d) complete histopathologic description of HCC; (e) MRI with sufficient image quality scanned within 1 month before surgery.

## Laboratory examinations and histopathology

Preoperative laboratory indexes (Table 1) comprised serum alpha-fetoprotein (AFP), carcinoembryonic antigen, carbohydrate antigen 19-9, des-gamma-carboxy prothrombin, hepatitis B virus (HBV), anti-hepatitis C virus, HBV-DNA loads, α-L-fucosidase, alanine aminotransferase (ALT), aspartate aminotransferase (AST), total bilirubin (TBIL), direct bilirubin, r-glutamyltransferase, alkaline phosphatase (AKP), total protein, albumin, platelet count, prothrombin time, activated partial thromboplastin time, fibrinogen, thrombin time, prealbumin, hyaluronic acid, procollagen type III, type IV collagen, and laminin. The Child-Pugh and Barcelona Clinic Liver Cancer (BCLC) stages were also included in this study.

**Table 1:** Clinical and radiologic hallmarks of the primary cohorts

Variables	Training cohort (*n* = 250)				Validation cohort (*n* = 106)				*p*_Inter_
	MVI-	MVI+	*p*_Intra_	OR (95% CI)	MVI-	MVI+	*p*_Intra_	OR (95% CI)	
Age, mean (SD), years	54.12 (12.26)	53.73 (9.89)	0.823	0.997 (0.973, 1.022)	54.51 (10.20)	55.07 (11.73)	0.900	0.913 (0.22, 3.792)	0.627
Sex (male/female)	165/25	50/10	0.496	1.320 (0.594, 2.934)	63/13	25/5	0.957	0.969 (0.313, 3.002)	0.470
BCLC (0/A stage)	101/89	22/38	0.027	1.960 (1.079, 3.562)	49/27	6/24	< 0.001	7.259 (2.643, 19.940)	0.643
Child-Pugh (A / B stage)	187/3	57/3	0.153	3.281 (0.644, 16.703)	74/2	29/1	0.845	1.276 (0.111, 14.617)	1.000
HBV or HCV^a^	24/166	7/53	0.840	1.095 (0.446, 2.684)	9/67	5/25	0.511	0.672 (0.205, 2.198)	0.834
HBV-DNA loads (≤ 10^4^/> 10^4^)	156/22	49/6	0.773	0.868 (0.333, 2.263)	62/10	24/3	0.716	0.775 (0.196, 3.061)	0.778
AFP (≤ 20, 20–400, > 400 ng/mL)	107/56/22	18/28/10	0.004	1.827 (1.211, 2.755)	48/25/3	12/7/9	0.003	2.640 (1.405, 4.959)	0.610
DCP (≤ 40/> 40 mAU/mL)	67/34	12/18	0.011	2.956 (1.277, 6.84)	43/23	8/14	0.021	3.272 (1.197, 8.942)	0.728
TBIL (≤ 20.4/> 20.4 μmol/L)	173/17	50/10	0.098	2.035 (0.877, 4.724)	66/10	24/6	0.379	1.650 (0.541, 5.030)	0.256
TP (≤ 65/> 65 g/L)^b^	46/144	21/39	0.102	0.593 (0.317, 1.109)	14/62	4/26	0.531	1.468 (0.441, 4.882)	0.047 ^b^
APTT (≤ 31.3/> 31.3 s)^b^	164/26	48/12	0.238	1.577 (0.741, 3.358)	71/5	27/3	0.551	1.578 (0.353, 7.060)	0.049 ^b^
FIB (≤ 200/> 200 mg/dL)^b^	58/132	23/37	0.261	0.707 (0.380, 1.295)	16/60	6/24	0.904	1.067 (0.373, 3.051)	0.027 ^b^
Other laboratory indexes	/	/	> 1.000		/	/	> 1.000		> 0.050
Tumor size (≤ 2/2–5 cm)	112/78	23/37	0.006	2.310 (1.274, 4.189)	51/25	7/23	< 0.001	6.703 (2.536, 17.717)	0.901
T1_HBP_, mean (SD)	456.94 (141.61)	528.39 (173.34)	0.007	1.003 (1.001, 1.005)	440.28 (123.51)	522.78 (205.95)	0.040	1.004 (1.000,1.008)	0.635
T1_pre_, mean (SD)	948.65 (260.27)	1020.73 (270.45)	0.079	1.001 (1.000, 1.002)	951.96 (286.40)	975.93 (230.20)	0.681	1.000 (0.999,1.002)	0.830
Edge roughness, mean (SD)	0.15 (0.09)	0.23 (0.15)	< 0.001	442.52 (22.78, 8597.07)	0.13 (0.07)	0.22 (0.12)	< 0.001	118262.81 (197.05, 70979058.40)	0.227
Typical MRI pattern^a^	21/169	6/54	0.819	1.118 (0.429, 2.914)	8/68	1/29	0.257	3.412 (0.408, 28.533)	0.819
Peritumoral enhancement ^a^	172/18	27/33	< 0.001	11.679 (5.781, 23.593)	68/8	15/15	< 0.001	6.317 (2.370, 16.843)	0.783
Peritumoral hypointensity^a^	180/10	36/24	< 0.001	12.0 (5.286, 27.244)	67/9	19/11	0.005	4.310 (1.558, 11.924)	0.205
Capsule enhancement (intact/incomplete/absent)	21/30/139	34/20/6	< 0.001	0.180 (0.116, 0.278)	13/6/57	9/13/8	0.001	0.408 (0.241, 0.689)	0.836

Other laboratory indexes: α-L-fucosidase (≤ 40/> 40 U/L), carcinoembryonic antigen (≤ 5/> 5 ng/mL), carbohydrate antigen 19–9 (≤ 34/> 34 ng/mL), albumin (≤ 35/> 35 g/L), direct bilirubin (≤ 6.8/> 6.8 umol/L), alanine aminotransferase (≤ 50/> 50 U/L), aspartate aminotransferase (≤ 40/> 40 U/L), alkaline phosphatase (≤ 125/> 125 U/L), r-glutamyltransferase (≤ 60/> 60 U/L), total bile acid (≤ 10/> 10 umol/L), platelet count (≤ 100 × 10^9^/L/> 100 × 10^9^/L), prothrombin time (≤ 13/> 13 s), thrombin time (≤ 21/> 21 s), hyaluronic acid (≤ 120/> 120 ng/mL), laminin (≤ 130/> 130 ng/mL), procollagen type III (≤ 15/> 15 ng/mL), type IV collagen (≤ 95/> 95 ng/mL)

*Abbreviations*: *OR*, odds ratio; *HBV*, hepatitis B virus; *HCV*, hepatitis C virus; *HBV-DNA*, deoxyribonucleic acid of hepatitis B virus; *AFP*, alpha-fetoprotein; *DCP*, des-gamma-carboxy prothrombin; *TBIL*, total bilirubin: *BCLC*, Barcelona Clinic Liver Cancer; *TP*, total protein; *APTT*, activated partial thromboplastin time; *FIB*, fibrinogen; *T1*_*PRE*_ and *T1*_*HBP*_, defined as the signal intensity of tumor derived from the pre-contrast and hepatobiliary phase T1 maps, respectively

^a^Absence/presence

*p*_*Intra*_: *p* value of univariate logistic regression analysis between the MVI+ and MVI− groups; *p*_*Inter*_: *p* value of the inter-cohort difference with chi-square test for categorical variables and independent samples *t* test for numeric variables

^b^*p*_Inter_ < 0.05: a significant difference between the training and validation cohorts, which was enrolled in the multivariate logistic regression analysis

HCC pathological samples were taken by a 7-point baseline sample collection protocol [12]. Histopathological characteristics (tumor size, number, Edmondson-Steiner grade, MVI status and category, liver fibrosis grade based on the Scheuer scoring system, and Ki-67 protein expression) were assessed in consensus by two experienced abdominal pathologists.

MVI was defined as the presence of tumor in the portal vein, hepatic vein, or a large capsular vessel of the surrounding hepatic tissue lined with endothelium, which was visible only on microscopy [7, 11, 12, 18]. According to the high-risk factors of adverse outcomes [12, 18, 19], the patients were classified into M0 (no MVI), M1 (invaded vessels were no more than five and located at the peritumoral region adjacent to the tumor surface within 1 cm), or M2 (MVI of > 5 or at > 1 cm away from the tumor surface) grades [12], respectively.

### Gd-EOB-DTPA MRI

MRI was performed at a 1.5-T scanner (Magnetom Aera, Siemens Healthcare) with intravenous bolus injection of 0.025 mmol/kg gadoxetate disodium (Primovist, Bayer Pharma). MRI sequences were as follows: axial T2-weighted imaging with fat suppression, DWI, in-phase and opposed-phase T1-weighted imaging (T1WI), pre-contrast three-dimensional volumetric-interpolated breath-hold (3D-VIBE) T1WI, post-contrast dynamic 3D-VIBE-T1WI (AP, 20–30 s; portal venous phase: PVP, 60–70 s; transitional phase: TP, 180 s; HBP: 20 min) after the injection of gadoxetate disodium, and automatically reconstructed pre-contrast and HBP T1 maps. Detailed parameters are shown in Table S1.

### Qualitative and quantitative analyses of MRI

Morphologic hallmarks (typical MRI pattern of HCC [1, 20], peritumoral enhancement [21], capsule enhancement [22], the longest diameter of tumor [23], and peritumoral hypointensity on HBP images [21]) were independently reviewed by two radiologists (S.X.R. and C.Y., 20 and 15 years of abdominal MRI experience) who were blinded to MVI status. Meanwhile, the average signal intensity of tumor on the pre-contrast and HBP T1 maps were measured and defined as T1_PRE_ and T1_HBP_, respectively. In case of any discrepancies, a consensus was reached after discussion. Instead of the subjective evaluation of tumor edge, “edge roughness” was automatically and quantitatively computed as the average distance from the actual tumor surface to its convex envelope on HBP images. Namely, edge roughness was a continuous value to measure the non-smoothness of tumor edge.

### Radiomics analysis

**Fig. 2:**
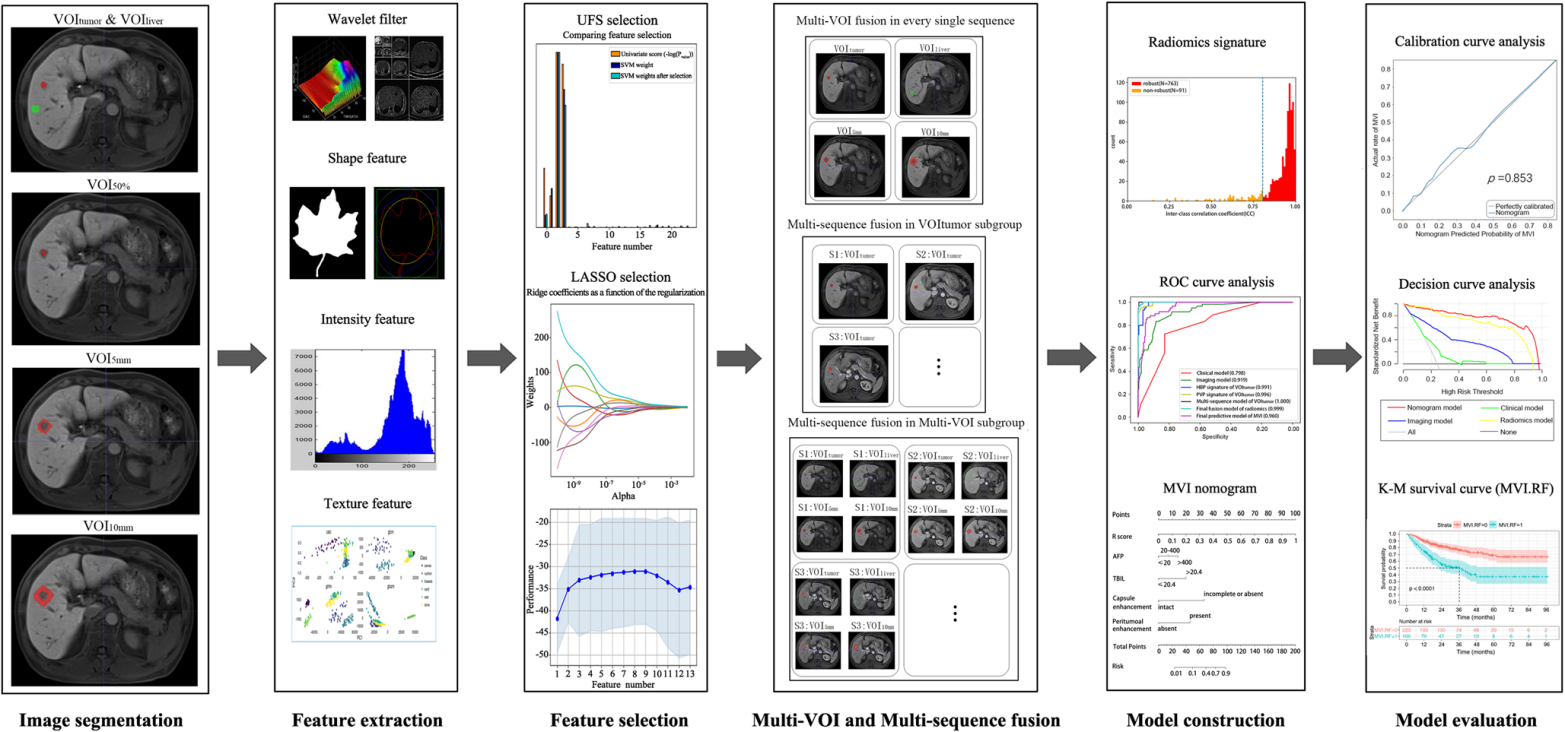
Flowchart of radiomics analysis

Radiomics was implemented by Python programming language (version 3.7.3, https://www.python.org) with Pyradiomics (version 2.2.0, https://pyradiomics.readthedocs.io/en/latest/index.html) and Scikit-learn (version 2.1.0, https://scikit-learn.org/stable/index.html) packages. Radiomics workflow comprised manual tumor segmentation, feature extraction and selection, multiple sequences and volumetric interests (VOIs) fusion, and model construction and evaluation (Fig. 2).

First, tumor boundaries were manually delineated on all single sequence images, denoted as VOI_tumor_, by two radiologists (H.H.C. and L.Y., 8 years and 10 years of abdominal imaging experiences) with ITK-SNAP software (http://www.itksnap.org/pmwiki/pmwiki.php). Besides, the two radiologists randomly picked 5 to 10 blocks distributed in different liver lobes sufficiently away from large vessels, artifacts, liver margins, and hepatic lesions, which were used as regions of normal liver tissues (VOI_liver_) for contrast analysis with tumor. To further explore the intratumoral and peritumoral information, the VOI_tumor_ was shrunk 50% (VOI_50%_) and dilated by 5 mm and 10 mm (VOI_5mm_ and VOI_10mm_) using standard image morphological erosion and dilation operations, respectively. Please note that VOI_5mm_ and VOI_10mm_ excluded the tumor region and only referred to the peritumoral zone within 5 mm and 10 mm from the tumor surface. Meanwhile, a variety of regional combinations were experimented, including VOI_tumor + liver_ which combined the tumor (VOI_tumor_) and the liver background (VOI_liver_) regions; VOI_tumor + 5mm_ and VOI_tumor + 10mm_ joined the tumor (VOI_tumor_) with peripheral zones VOI_5mm_ and VOI_10mm_, respectively, based on which VOI_tumor + 5mm + liver_ or VOI_tumor + 10mm + liver_ was defined with additional VOI_liver_ merged.

Subsequently, a set of 854 features radiomics features were extracted from the original and three-dimensional wavelet filters images [24], including tumor shape, size, intensity, and texture (Table S2). These features were first selected by the Least Absolute Shrinkage and Selection Operator (LASSO, Table S3) for each VOI of each single sequence. The first selected features were then combined to obtain the optimal multi-VOI models in single sequences (Tables 2 and S4). These multi-VOI features of each sequence were finally joined and selected using LASSO again (Table S5) to derive the ultimate multi-sequence, multi-VOI radiomics model (Table S6), and based on which MVI nomograms were constructed with random forest (RF) and logistic regression (LR) classifiers respectively for comparison. Finally, the receiver operating characteristic, calibration, and decision curves were plotted and the validation data was tested for model evaluation.

**Table 2:** Results of single sequences based on multiple volumetric interests for predicting MVI

Sequence	Classifier and cohort	AUC						
		VOI_50%_	VOI_tumor_	VOI_tumor + 5mm_	VOI_tumor + 10mm_	VOI_tumor + liver_	VOI_tumor + 5mm + liver_	VOI_tumor + 10mm + liver_^a^
T2WI	RF (TD/VD)	0.818/0.722	0.832/0.714	0.897/0.730	0.816/0.742	0.841/0.726	0.867/0.749	0.975/*0.755*
	LR (TD/VD)	0.641/0.698	0.647/0.708	0.632/0.725	0.650/0.712	0.647/0.708	0.632/0.725	0.638/*0.727*
DWI	RF (TD/VD)	0.830/0.736	0.980/0.778	0.879/0.793	0.828/0.791	0.813/0.784	0.832/0.793	0.978/*0.812*
	LR (TD/VD)	0.695/0.701	0.752/0.703	0.663/0.775	0.655/0.777	0.681/0.731	0.664/0.774	0.667/*0.780*
PRE	RF (TD/VD)	0.829/0.737	0.938/0.765	0.898/0.771	0.813/0.761	0.991/0.782	0.878/0.797	0.912/*0.810*
	LR(TD/VD)	0.746/0.749	0.730/0.752	0.728/0.757	0.728/0.757	0.730/0.761	0.730/0.773	0.735/*0.782*
Pre-T1 maps	RF (TD/VD)	0.802/*0.793*	0.720/0.714	0.642/0.738	0.669/0.758	0.677/0.717	0.826/0.740	0.752/0.766
	LR(TD/VD)	0.633/*0.765*	0.658/0.724	0.643/0.714	0.631/0.746	0.648/0.715	0.652/0.714	0.637/0.754
AP	RF (TD/VD)	0.980/0.685	0.873/0.765	1.000/0.812	0.996/0.802	0.948/0.777	0.886/0.815	0.944/*0.830*
	LR (TD/VD)	0.701/0.692	0.715/0.693	0.686/0.746	0.731/0.742	0.639/0.719	0.821/0.761	0.715/*0.761*
PVP	RF (TD/VD)	0.920/0.740	0.996/0.810	0.876/0.832	0.808/0.818	0.902/0.825	0.902/0.836	0.912/*0.837*
	LR (TD/VD)	0.761/0.706	0.755/0.768	0.728/0.798	0.731/0.799	0.733/0.796	0.732/0.800	0.727/*0.806*
TP	RF (TD /VD)	0.900/0.729	0.963/0.728	0.995/0.738	0.854/0.778	0.884/0.749	0.871/0.762	0.802/*0.792*
	LR (TD /VD)	0.716/0.683	0.718/0.716	0.720/0.707	0.739/0.754	0.720/0.725	0.736/0.720	0.751/*0.762*
HBP	RF (TD/VD)	0.712/0.784	0.991/0.799	0.874/0.831	0.976/0.789	1.000/0.808	0.866/0.827	0.885/*0.855*
	LR (TD/VD)	0.676/0.723	0.744/0.746	0.678/0.735	0.770/0.759	0.743/0.762	0.751/0.803	0.715/*0.805*
HBP-T1 maps	RF (TD/VD)	0.923/0.718	0.808/0.705	0.821/0.726	0.821/0.726	0.822/0.724	0.822/0.729	0.807/*0.731*
	LR (TD/VD)	0.705/0.703	0.706/0.703	0.691/0.708	0.684/0.715	0.683/0.714	0.705/0.715	0.702/*0.716*

*Abbreviations*: *VOI*, volumetric interest; *AUC*, area under the curve; *VD*, validation dataset; *TD*, training dataset; *RF*, random forest; *LR*, logistic regression; *T2WI*, T2-weighted imaging with fat suppression; *DWI*, diffusion-weighted imaging; *PRE*, pre-contrast phase; *AP*, arterial phase; *PVP*, portal venous phase; *TP*, transitional phase; *HBP*, hepatobiliary phase

^a ^The sensitivity, specificity, and AUC of VOI_tumor + 10mm + liver_ using random forest in each single sequence for predicting histologic MVI are listed in Table S4

Italicized values indicated the highest AUC of validation cohort in each single sequence

### Outcome analysis

Follow-up was performed at intervals of 3 to 6 months after curative surgery. The date of surgery, recurrence, metastasis, death, and the last follow-up were recorded for calculating the overall and recurrence-free survival (RFS).

### Statistical analyses

Statistical analyses were performed with IBM SPSS Statistics (version 25) and R (version 3.6.1, https://www.r-project.org) software. Patients enrolled in MVI or outcome study were randomly allocated to training and validation cohorts in a ratio of 7:3. The discrimination performance of models was quantified by area under the curve (AUC) and net reclassification index (NRI). NRI > 0 meant a positive improvement, indicating that the predictive ability of the new model precedes the old one. Compared to the histologic MVI, the preoperatively predicted MVI status was calculated by MVI nomogram using RF (MVI-RF) or LR (MVI-LR) in each patient, with prediction probabilities > 50% classified into MVI-positive group and > 90% defined as M2 grade. *P* < 0.05 was considered statistically significant.

More details (T1 maps and morphologic hallmarks, feature extraction and selection, and statistical analyses) are available in the Supplementary Materials and Methods.

## Results

### Clinicoradiologic characteristics and performances for predicting MVI

Among the 356 solitary HCC patients, only 90 patients suffered from MVI, 347 patients underwent hepatectomy, and 9 patients received liver transplantation. The univariate LR results of clinicoradiologic characteristics are summarized in Table 1. In multivariate LR analysis of the training cohort, AFP > 20 ng/mL (*p* = 0.006, OR = 7.683, 95% CI: 1.776–33.245) and TBIL > 20.4 μmol/L (*p* = 0.010, OR = 8.420, 95% CI: 1.658–42.766) were independent risk factors for MVI in the clinical model.

**Fig. 3:**
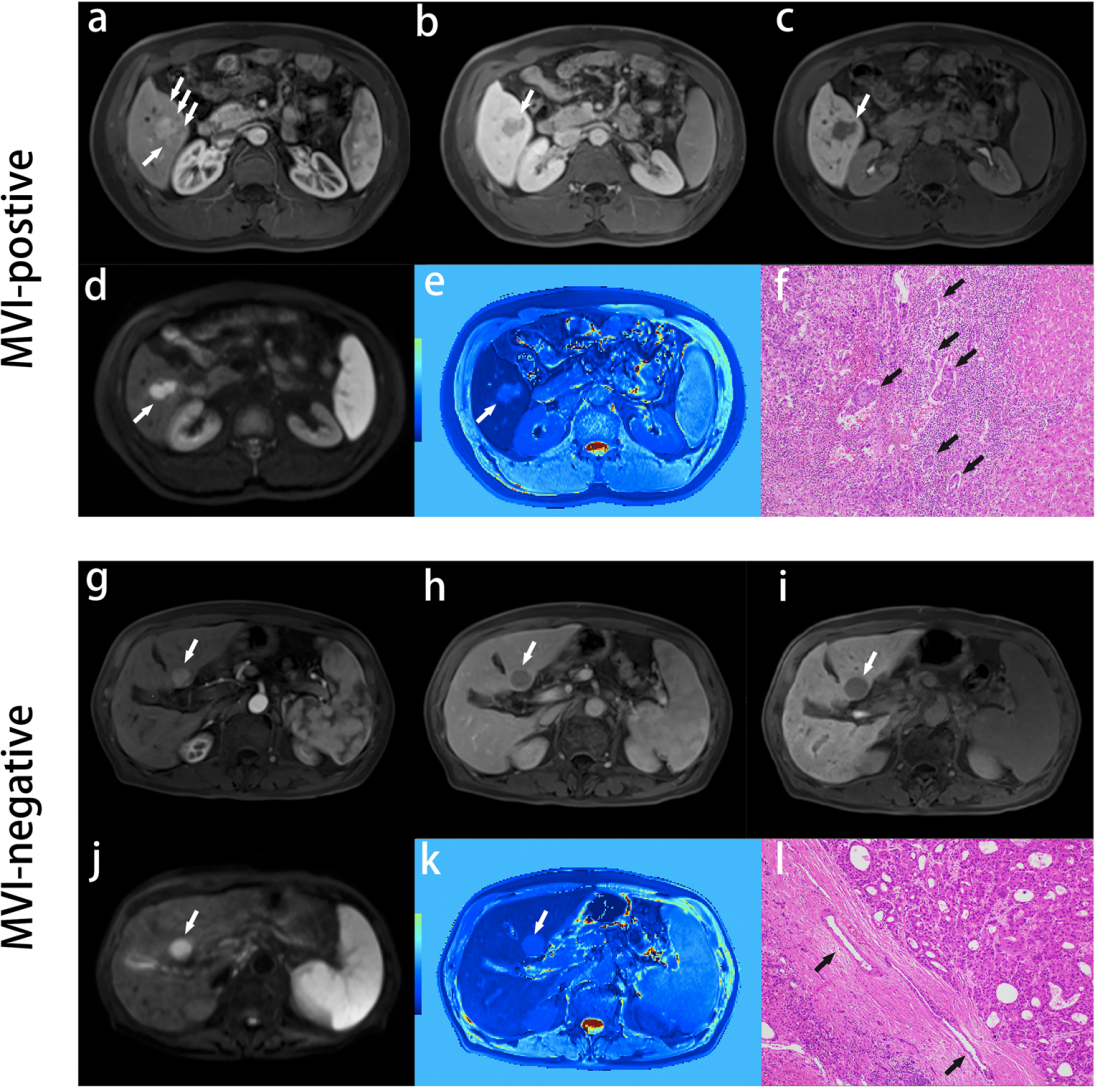
Representative images of MVI-positive and MVI-negative patients. MVI-positive case: A 51-year-old male with elevated AFP, TBIL, and AKP levels (320 ng/mL, 32.6 μmol/L, and 131 U/L) was admitted to our department for abdominal discomfort and yellow sclera and identified intrahepatic recurrence at 11 months after hepatectomy. Gd-EOB-DTPA MRI detected a solid lesion (2.9 × 1.9 cm) in hepatic segment V, with the architectures of wedge-shaped peritumoral enhancement on arterial phase images (**a**, arrows), absent capsule enhancement on transitional phase images (**b**, arrows), non-smooth tumor edge on HBP, DWI, and HBP T1 maps (**c**–**e**, arrows), and typical MRI pattern of HCC (non-rim arterial phase enhancement and non-peripheral transitional phase hypointensity). M2 grade was diagnosis by postoperative pathological specimens with standard hematoxylin and eosin (HE, × 100): multiple tumor thrombi of microvasculature (**f**, black arrow; MVI > 5) were distributed in the widespread inflammatory cells, which were located at the region between the normal liver tissue in the right side and the infiltrating HCC lesion without tumor capsule in the upper left corner. MVI-negative case: A 77-year-old male with normal levels of AFP, TBIL, and AKP (3.4 ng/mL, 11.7 μmol/L, and 90 U/L) was admitted to our hospital for a liver lesion in health examination, and identified recurrence-free until April 2020 (18 months after hepatectomy). Gd-EOB-DTPA MRI detected a well-circumscribed solid lesion (2.3 × 2.0 cm) in hepatic segment II, with the architectures of absent peritumoral enhancement (**g**, arrows), intact capsule enhancement (**h**, arrows), smooth tumor margin (**i**–**k**, arrow), and typical MRI pattern of HCC. M0 grade was diagnosed by pathologic HE (× 100) sample: no tumor thrombus was detected in microvascular system (**l**, black arrow), which were located at the region between the normal liver tissue in the lower left corner and the HCC lesion with intact capsule in the upper right corner

Edge roughness was significantly different between MVI-negative and MVI-positive patients (0.147 ± 0.080 vs 0.224 ± 0.137, *p* < 0.001), indicating higher value of edge roughness (less spheroid of tumor) was positively correlated with MVI. In multivariate LR analysis, absent or incomplete capsule enhancement (*p* < 0.001, OR = 18.678, 95% CI: 6.129–56.925), higher value of edge roughness (*p* = 0.05, OR = 68.886, 95% CI: 0.974–4874.172), and peritumoral enhancement (*p* < 0.001, OR = 5.721, 95% CI: 2.161–15.151) were independent risk factors for MVI in the imaging model. Representative MVI images are shown in Fig. 3. The MVI predictive performances of clinical and imaging models are summarized in Table 3.

**Table 3:** The performance of the clinical, imaging, radiomics model and the nomogram for predicting MVI

Models	Classifier	Training cohort (*n* = 205)			Validation cohort (*n* = 106)			Cutoff
		Sen	Spe	AUC (95% CI)	Sen	Spe	AUC (95% CI)	
Clinical	RF	0.72	0.83	0.798 (0.739–0.857)	0.73	0.59	0.725 (0.647–0.803)	0.25
	LR	0.73	0.72	0.779 (0.719–0.837)	0.70	0.55	0.668 (0.570–0.766)	0.17
Imaging	RF	0.83	0.88	0.919 (0.880–0.958)	0.77	0.87	0.876 (0.816–0.934)	0.31
	LR	0.82	0.84	0.894 (0.855–0.933)	0.83	0.67	0.792 (0.713–0.869)	0.13
Radiomics ^a^	RF	1.00	0.97	0.999 (0.999–0.999)	0.96	0.86	0.918 (0.859–0.977)	0.26
	LR	0.70	0.69	0.773 (0.714–0.832)	0.63	0.88	0.809 (0.731–0.887)	0.27
Nomogram	RF	0.87	0.94	0.960 (0.940–0.980)	0.93	0.85	0.920 (0.861–0.979)	0.23
	LR	0.92	0.84	0.934 (0.895–0.973)	0.93	0.75	0.879 (0.820–0.938)	0.19

*Abbreviations*: *RF*, random forest; *LR*, logistic regression; *Sen*, sensitivity; *Spe*, specificity; *AUC*, area under the curve; *CI*, confidence interval

Radiomics ^a^: the final radiomics model based on the multi-parametric (arterial phase, portal venous phase, hepatobiliary phase T1-weighted image, and diffusion-weighted imaging) fusion in VOI_tumor + 10mm + liver_

### Performance of radiomics features from single sequences

The AUCs of each VOI in single sequences are displayed in Table 2. For the vast majority of VOIs and sequences, RF outperformed LR classifier, HBP, and PVP were superior to other sequences, and the VOI_tumor + 10mm + liver_ yielded the best multi-VOI fusion for predicting MVI. Interestingly, the AUCs of VOI_50%_, VOI_tumor_, VOI_tumor + 5mm_, VOI_tumor + 10mm,_ and VOI_tumor + 10mm + liver_ subgroups approximately kept increasing almost in all sequences regardless of the choice of classifiers. Notably, VOI_tumor + 10mm + liver_ showed consistent performance improvements compared to VOI_tumor_ in HBP and PVP sequences on the validation cohort (NRIs > 0, Table 4).

**Table 4:** Net reclassification indexes and *p* values of diverse combinations

Subgroups	Diverse combinations	Classifier and cohort	NRI (%)	*p* (NRI)	*p* (AUC)
Single sequence	VOI_tumor + 10mm + liver_ vs. VOI_tumor_ on HBP	RF (TD/VD)	- 31.03%/17.70%	1.000/0.072	0.960/0.313
		LR (TD/VD)	- 10.34%/6.44%	0.971/0.169	0.700/0.245
	VOI_tumor + 10mm + liver_ vs. VOI_tumor_ on PVP	RF (TD/VD)	- 24.35%/7.81%	1.000/0.187	0.915/0.371
		LR (TD/VD)	- 3.38%/0.44%	0.770/0.486	0.672/0.334
VOI_tumor_	Multi-parametric ^a^ vs. HBP	RF (TD/VD)	5.68%/19.28%	0.002/*0.046*	0.441/0.206
		LR (TD/VD)	10.96%/3.24%	0.049/0.408	0.134/0.294
	Multi-parametric ^a^ vs. PVP	RF (TD/VD)	6.49%/20.90%	0.021/*0.017*	0.467/0.238
		LR (TD/VD)	2.76%/1.35%	0.307/0.410	0.165/0.393
VOI_tumor + 10mm + liver_	Multi-parametric ^b^ vs. HBP	RF (TD/VD)	35.04%/19.44%	< 0.001/*0.008*	0.030/0.192
		LR (TD/VD)	11.14%/3.24%	0.031/0.391	0.173/0.482
	Multi-parametric ^b^ vs. PVP	RF (TD/VD)	27.99%/24.54%	< 0.001/*0.003*	0.075/0.180
		LR (TD/VD)	- 0.11%/4.63%	0.507/0.349	0.229/0.486
Model	Radiomics vs. Clinical model	RF (TD/VD)	41.8%/54.1%	0.001/*0.002*	0.012/*0.050*
		LR (TD/VD)	- 16.1%/11.1%	0.856/0.298	0.527/0.116
	Radiomics vs. Imaging model	RF (TD/VD)	25.7%/22.2%	< 0.001/*0.029*	0.095/0.321
		LR (TD/VD)	- 26.1%/2.3%	0.997/0.442	0.977/0.426
	Nomogram vs. Clinical model	RF (TD/VD)	19.7%/56.8%	0.091/**<** *0.001*	0.004/*0.013*
		LR (TD/VD)	14.6%/47.7%	0.133/*0.005*	0.005/*0.009*
	Nomogram vs. Imaging model	RF (TD/VD)	9.1%/14.0%	0.038/0.075	0.249/0.309
		LR (TD/VD)	9.5%/78.9%	0.070/*0.041*	0.254/0.163
	Nomogram vs. Radiomics model	RF (TD/VD)	- 16.3%/- 2.8%	0.999/0.647	0.790/0.491
		LR (TD/VD)	35.7%/19.4%	< 0.001/0.054	0.004/0.217

Net reclassification index (NRI): NRI > 0 was a positive improvement, indicating that the predictive ability of the new model was better than the old one

*Abbreviations*: *AUC*, area under curve; *VD*, validation dataset; *TD*, training dataset; *RF*, random forest; *LR*, logistic regression

Multi-parametric ^a^: the best combination (portal venous phase, hepatobiliary phase, arterial phase T1-weighted image, and pre-contrast T1 map) in the VOI_tumor_ subgroup

Multi-parametric ^b^ or Radiomics model: the optimal radiomics model based on the best combination (portal venous phase, hepatobiliary phase, arterial phase T1-weighted image, and diffusion-weighted imaging) in the VOI_tumor + 10mm + liver_ subgroup

Italicized values: *p* < 0.05 in the validation cohort

### Performance of radiomics features from multiple sequences

**Fig. 4:**
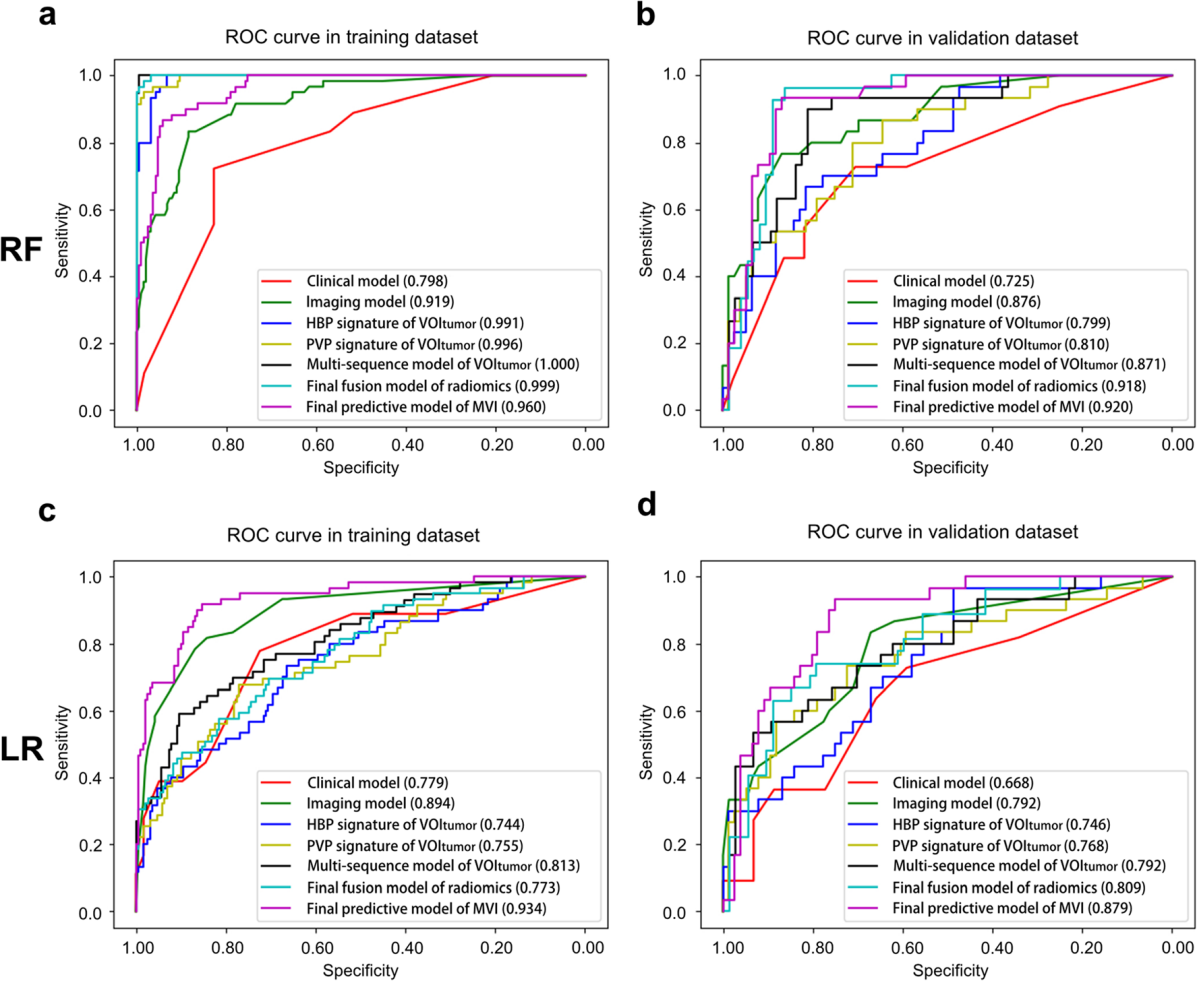
Receiver operating characteristic curves of different models for predicting MVI. Receiver operating characteristic curves of different models for predicting MVI were plotted by random forest (**a**: training cohort, **b**: validation cohort) and logistic regression (**c**: training cohort, **d**: validation cohort) to crossly validate the robustness of models

In the VOI_tumor_ subgroup, the MVI predictive efficacies of two best single sequences (HBP and PVP) were worse than any of the multi-sequence models either using RF or LR (Table S7), especially inferior to that of the best combination (PVP, HBP, AP, and pre-contrast T1 maps; AUCs of validation cohort: 0.871 using RF and 0.792 using LR; Fig. 4). Concretely, this four-sequence model showed significant improvements compared to the two best single sequences (HBP: NRI 19.28%, *p* = 0.046; PVP: NRI 20.90%, *p* = 0.017; Table 4 ) in the validation cohort using RF.

In the VOI_tumor + 10mm + liver_ subgroup, the optimal multi-sequence fusion was the integration of PVP, HBP, AP, and DWI, with AUCs of 0.918 using RF and 0.809 using LR in the validation cohort (Tables 3 and S6). Meanwhile, the predictive performance of this four-sequence fusion—the final radiomics model—was also significantly superior to those of the two best single sequences (HBP: NRI 19.44%, *p* = 0.008; PVP: NRI 24.54%, *p* = 0.003; Table 4) as well as those of the clinical (NRI 54.1%, *p* = 0.002) and imaging models (NRI 22.2%, *p* = 0.029) using RF in the validation cohort. The details of the top six most discriminating features in the final radiomics model are provided in Table S8.

### Performance of MVI nomograms

**Fig. 5:**
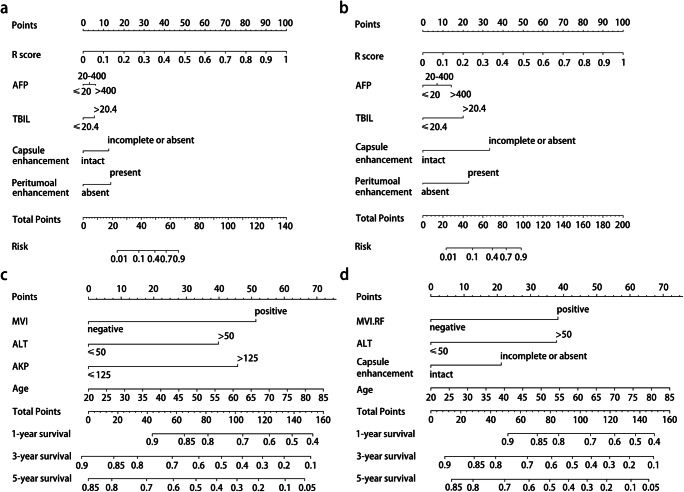
Nomograms for predicting MVI and recurrence-free survival. The final predictive model of MVI was visualized as nomograms (**a**: random forest, **b**: logistic regression). The independent predictors of recurrence were graphically shown as nomograms in the histologic MVI (**c**) and the predicted MVI-RF (**d**) subgroups, respectively

Based on the clinical, imaging, and final radiomics predictors, the ultimate MVI predictive model incorporated the independent risk factors of TBIL > 20.4 μmol/L, AFP > 20 ng/mL, incomplete or absent capsule enhancement, peritumoral enhancement, and higher score of radiomics (R-score) into visualized nomograms (Fig. 5a–b) as follows:

- the nomogram using RF:
  $$ Y=-8.38+16.13\times R\  score+2.04\times \mathrm{capsule}\ \mathrm{enhancement}+2.20\times \mathrm{peritumoral}\ \mathrm{enhancement}+0.90\times \mathrm{TBIL}+0.50\times \mathrm{AFP} $$
- the nomogram using LR:
  $$ Y=-6.70+9.07\times \mathrm{R}\ \mathrm{score}+3.02\times \mathrm{capsule}\ \mathrm{enhancement}+2.07\times \mathrm{peritumoral}\ \mathrm{enhancement}+1.83\times \mathrm{TBIL}+0.64\times \mathrm{AFP} $$

The predictive performances of MVI nomograms (Tables 3 and 4) demonstrated moderately or dramatically enhancements compared to those of clinical models (NRIs: 14.6–56.8%), imaging models (NRIs: 9.1–78.9%), and radiomics model using LR (NRIs: 19.4–35.7%), with a slightly negative improvement contrasted to radiomics model using RF (NRI < 0, *p* > 0.05). Furthermore, the AUCs (Fig. 4) of HBP sequence in VOI_tumor_, PVP sequence in VOI_tumor_, multi-sequence fusion in VOI_tumor_, the final radiomics in VOI_tumor + 10mm + liver_, and the ultimate predictive model of MVI (MVI nomogram) presented a gradual upward trend in validation cohort using RF or LR classifier. Being highly consistent with the actual MVI status in the calibration curves (Fig. S1a–d), MVI nomograms obtained the best net clinical benefit, followed by the radiomics and imaging models, with the clinical model worst in the decision curves (Fig. S1e–h).

### Clinical outcome

Until April 2020, 329 patients had completed follow-up. While 1-, 3-, and 5-year overall survival rates reached 99.0%, 95.4%, and 93.6%, 1-, 3-, and 5-year RFS rates were 85.4%, 72%, and 68.4%, respectively. Therefore, this study only focused on the RFS analysis.

**Fig. 6:**
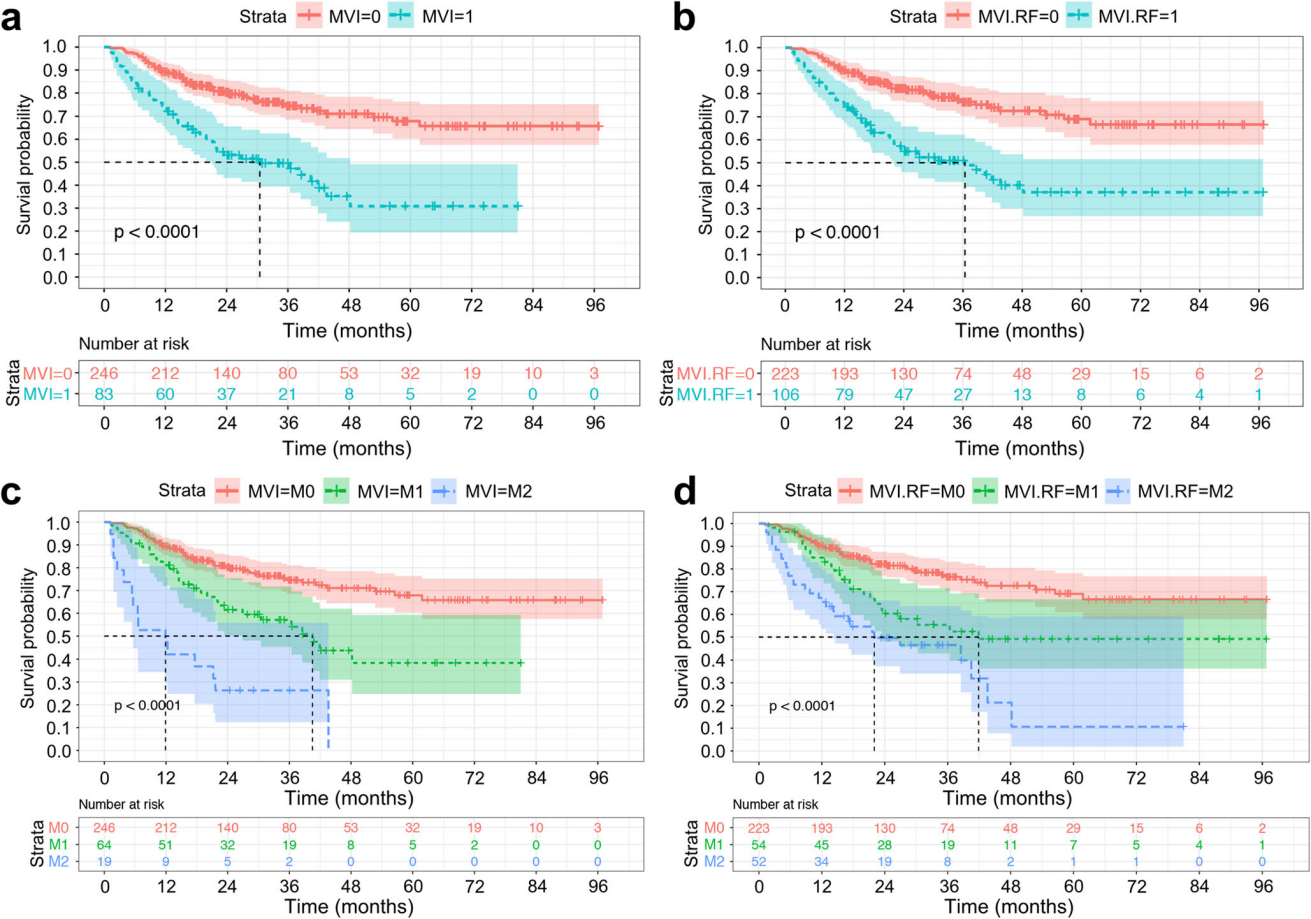
Kaplan-Meier curves of recurrence-free survival. With the Kaplan-Meier analysis and 2-sided log-rank test, recurrence-free survival curves were scaled by the histologic MVI status (**a**) and the predicted MVI status (**b**) by MVI nomogram using random forest (MVI-RF) and were further stratified by the histologic MVI (**c**) and MVI-RF grades (**d**), respectively

The median RFS of histologic MVI-positive (M2, M1) and MVI-negative (M0) patients were 30.5 months (M2, 11.9 months; M1, 40.5 months) and > 96.9 months (log-rank test, *p* < 0.001, Fig. 6), respectively. Similarly, the median RFS of positive and negative MVI-RF groups were 36.4 months (M2, 22.0 months; M1, 41.9 months) and > 96.9 months (*p* < 0.001), respectively. However, MVI-LR failed to satisfy the proportional hazard assumption in the discrimination and stratification of MVI for predicting RFS (log-rank test, *p* = 0.735, 0.224; Fig. S2).

The results of multivariate Cox regression (Table 5) presented that histologic MVI, AKP > 125 U/L, ALT > 50 U/L, and the elderly were independent risk factors for recurrence, with C-indexes of 0.704 (95% CI: 0.645–0.764; *n* = 230) in the training cohort and 0.654 (95% CI: 0.538–0.769; *n* = 99) in the validation cohort. Similarly, the positive MVI-RF status, incomplete or absent capsule enhancement, ALT > 50 U/L and the elderly were independent risk factors of recurrence in MVI-RF subgroup, with C-indexes of 0.700 (95% CI: 0.638–0.763) in the training cohort and 0.673 (95% CI: 0.570–0.776) in the validation cohort. The RFS nomograms of the histologic and predicted MVI subgroups are showed in Fig. 5c, d, and their calibration curves are available in Fig. S3.

**Table 5:** Variables associated with recurrence-free survival according to the Cox proportional hazards model

Variables	Univariate analysis		Multivariate analysis (histologic MVI subgroup)		Multivariate analysis (predicted MVI-RF subgroup)	
	*p*	HR (95% CI)	*p*	HR (95% CI)	*p*	HR (95% CI)
Age	0.020	1.025 (1.004, 1.046)	0.044	1.022 (1.001, 1.044)	0.047	1.022 (1.000, 1.044)
Ki-67	0.077	1.010 (0.999, 1.021)				
Cirrhosis^a^	0.505	1.175 (0.731, 1.887)				
ES (III–IV/I–II)	0.085	1.489 (0.946, 2.343)				
HBV or HCV ^a^	0.076	0.581 (0.319, 1.058)				
LD (2–5 cm / ≤ 2 cm)	0.922	1.023 (0.650, 1.608)				
Histologic MVI ^a^	< 0.001	2.703 (1.702, 4.293)	< 0.001	2.733 (1.712, 4.362)	/	/
Predicted MVI-RF ^a^	< 0.001	2.593 (1.652, 4.072)	/	/	< 0.001	2.258 (1.416, 3.601)
BCLC (A/0 stage)	0.622	1.120 (0.714, 1.758)				
Child-Pugh (B/A class)	0.009	3.382 (1.362, 8.396)				
Alpha-fetoprotein (> 20/≤ 20 ng/ml); (> 400/≤ 400 ng/ml)	0.261 0.878	1.302 (0.822, 2.064); 1.051 (0.554, 1.997)				
ALB (≤ 35/> 35 g/L)	0.038	0.295 (0.093, 0.937)				
AST (> 40/≤ 40 U/L)	0.072	1.627 (0.958, 2.766)				
GGT (> 60/≤ 60 U/L)	0.016	1.787 (1.117, 2.860)			0.058	1.614 (0.984, 2.648)
ALT (> 50/≤ 50 U/L)	0.001	2.528 (1.484, 4.305)	0.003	2.241 (1.307, 3.843)	0.012	2.067 (1.176, 3.635)
TBA (> 10/≤ 10 umol/L)	0.091	1.485 (0.939, 2.350)				
AKP (> 125/≤ 125 U/L)	0.023	2.653 (1.145, 6.149)	0.045	1.022 (1.001, 1.044)		
Ascites ^a^	0.008	2.714 (1.302, 5.656)				
Typical MRI pattern^a^	0.698	1.180 (0.512, 2.723)				
Edge non-smoothness	0.087	5.868 (0.755, 44.428)				
Capsule enhancement ^b^	0.002	1.861 (1.265, 2.739)			0.041	1.662 (1.021, 2.706)
Peritumoral enhancement ^a^	0.001	1.995 (1.319, 3.015)				
Peritumoral hypointensity ^a^	< 0.001	2.330 (1.510, 3.595)				
Other indexes	> 0.100					

Other indexes: sex (male/female), α-L-fucosidase (≤ 40/> 40 U/L), carcinoembryonic antigen (≤ 5/> 5 ng/mL), carbohydrate antigen 19–9 (≤ 34/> 34 ng/mL), platelet count (≤ 100 × 10^9^/L/> 100 × 10^9^/L), total bilirubin (≤ 20.4/> 20.4 μmol/L), direct bilirubin (≤ 6.8/> 6.8 umol/L), total protein (≤ 65/> 65 ng/mL), prealbumin (≤ 180/> 180 mg/L), hyaluronic acid (≤ 120/> 120 ng/mL), procollagen type III (≤ 15 /> 15 ng/mL), type IV collagen (≤ 95/> 95 ng/mL), laminin (≤ 130/> 130 ng/mL), prothrombin time (≤ 13/> 13 s), activated partial thromboplastin time (≤ 31.3/> 31.3 s), fibrinogen (≤ 200/> 200 mg/dlL), thrombin time (≤ 21/> 21 s); portal hypertension (present/absent); *T1*_*PRE*_ *and T1*_*HBP*_, defined as the signal intensity of tumor derived from the pre-contrast and hepatobiliary phase T1 maps, respectively

*Abbreviations*: *ES*, Edmondson-Steiner grades; *LD*, the longest diameter of tumor; *BCLC*, Barcelona Clinic Liver Cancer: *TBA*, total bile acids; *ALT*, alanine aminotransferase; *AST*, aspartate aminotransferase; *ALB*, albumin; *AKP*, alkaline phosphatase; *GGT*, r-glutamyltransferase; *HR*, hazard ratio;*CI*, confidence interval

^a ^Present/absent; ^b ^Incomplete-absent/intact capsule enhancement

## Discussion

Our study developed radiomics-based nomograms for preoperatively predicting MVI and outcome in patients with solitary HCC ≤ 5 cm. The results demonstrated that AFP > 20 ng/mL, TBIL > 20.4 μmol/L, absent or incomplete capsule enhancement, peritumoral enhancement, and higher R-score were independent risk factors for MVI. Mainly based on radiomics signatures of PVP, HBP, AP, and DWI in VOI_tumor + 10mm + liver_, the nomogram using RF or LR excellently identified MVI-positive patients. Furthermore, histologic MVI, ALT > 50 U/L, AKP > 125 U/L, and the elderly independently impaired RFS, with a relatively favorable prediction for recurrence. Histologic M0, M1, and M2 grades were significantly inverse correlated with RFS. Intriguingly, contrasted to histologic MVI, MVI-RF achieved comparable accuracy in MVI stratification and prognostic analyses.

Elevated AFP level [7, 11, 16], incomplete capsule enhancement [7, 8], and peritumoral enhancement [8, 9, 11] have been reported to be independent risk factors for MVI, which are consistent with our results. Independently facilitating MVI in this study, elevated TBIL level may secondary to the existence or obstruction of MVI in the biliary system [12, 18, 25]. This is partly due to the fact that cancerous thrombus in the newly formed bile ducts of tumor capsule [26], bile canaliculus, or interlobular bile ducts, rather than in gross or intrahepatic bile ducts, are difficult to be identified by preoperative imaging and excluded from the study population.

Peritumoral tissue is the first and most frequently vulnerable to MVI [21, 27], the vessels of which further serve as the main hematogenous dissemination pathway of portal vein tumor thrombosis and metastasis [21]. Therefore, we constructed multi-VOI models for exploring this highly aggressive region. Interestingly, the AUCs of VOI_50%_, VOI_tumor_, VOI_tumor + 5mm_, VOI_tumor + 10mm_, and VOI_tumor + 10mm + liver_ signatures approximately kept increasing almost in all sequences irrespective of classifiers. These preponderances of VOI_tumor_ over VOI_50%_ and VOI_tumor + 10mm_ over VOI_tumor_ for predicting MVI were consistent with the CT results of Xu et al [8] and HBP results of Feng et al [13], respectively. Meanwhile, the AUCs of VOI_tumor_, VOI_tumor + liver_ and VOI_tumor + 5mm + liver_ (VOI_tumor + 10mm + liver_) features, as well as those of VOI_tumor_, VOI_tumor + 5mm_ (VOI_tumor + 10mm_) and VOI_tumor + 5mm + liver_ (VOI_tumor + 10mm + liver_) signatures, also showed an increasing trend. Notably, the performance of VOI_tumor + 10mm + liver_ signatures preceded that of VOI_tumor + 10mm_ features either in this paper or in Feng et al study [13]. Besides, the optimal multi-sequence fusion outperformed the two best single sequences both in VOI_tumor_ and in VOI_tumor + 10mm + liver_ subgroups. These results signified the superiority of tumor periphery compared with tumor interior, the significance of texture and intensity difference between normal liver and intra-/peritumoral tissue, and the synergistic effect of multi-sequence and multi-VOI fusion for predicting MVI, which have been neglected in and might be the reason why our MVI nomograms obtained better performances than previous radiomics studies [8, 11, 13, 15, 16, 28].

Likewise, the top 6 most discriminating signatures of the final radiomics model also indicated the importance of peritumoral and intratumoral fusion. Being partly coincided with previous studies [8, 11], the six signatures included tumor size, shape, and intratumoral and peritumoral texture heterogeneity. By definition, HBP_VOI_5mm__wavelet-HHL_firstorder_Energy and HBP_VOI_5mm__wavelet-HLL_glszm_GrayLevelNonUniformity involved the texture heterogeneity of the peritumoral tissue within 5 mm, which might reflect an aggressive tendency to invade the tumor capsule and protrude into the peritumoral non-neoplastic parenchyma [27]. In addition, HBP_VOI_tumor__original_shape_Sphericity and DWI_VOI_tumor__original_shape_MajorAxisLength represented the spherical disproportion and the largest axis length of tumor, respectively. These were analogue to the well-known independent hallmarks “non-smooth edge and the longest diameter of tumor” of MVI [7–9, 11]. Furthermore, HBP_VOI_tumor__wavelet-HLL_glszm_SizeZoneNonUniformity and HBP_VOI_tumor__original_glszm_GrayLevelNonUniformity concerning intratumoral texture heterogeneity might be induced by tumor cellularity, micronecrosis and inflammation, for which further facilitated MVI [11, 29]. Coincidentally, five-sixths features were extracted from HBP, suggesting the significance of Gd-EOB-DTPA MRI in MVI diagnosis.

Histologic MVI [8, 10, 30], the elderly [30–32], incomplete or absent capsule enhancement [33, 34], and elevated ALT [30–32] and AKP [35–37] levels have been reported to impair outcomes of HCC patients, which were corresponded to our results. Conforming to the outcomes of few studies with histologic MVI grades [19, 38], our histologic MVI stratification, especially the novel and non-invasive MVI-RF classifications, showed significantly inverse correlations with RFS. Hence, the MVI-RF—an excellent substitute of histologic MVI—may be employed in patients with solitary HCC ≤ 5 cm, especially for those who underwent ablation without histologic MVI data. Namely, MVI-positive or even M2-grade patients diagnosed by MVI-RF before ablation might require more active clinical treatment and intense follow-up. Nevertheless, the AUCs of RFS nomograms around 0.66 for histologic MVI and MVI-RF subgroups, the unsatisfactory results may be induced by (1) the paucity of postoperative characteristics (e.g., preventive transarterial chemoembolization, immunosuppression therapy); (2) the absence of robust radiomics analysis in terms of recurrence instead of MVI; (3) the exclusion of well-established key predictors of recurrence (e.g., tumor size beyond 5 cm, satellite nodules or multifocal HCC, cancerous thrombus in gross bile ducts or vessels) in our study population.

This study has several limitations. Firstly, this paper is a retrospective single-centre study in China and needs to be validated by the external cohort. Secondly, we did not incorporate genomics with radiologic hallmarks, just as Banerjee et al [39]. Thirdly, this study focused on the solitary HCC within 5 cm, leading to a slightly lower frequency of MVI in our population than those of previous MVI studies with macrovascular invasive, larger, or multifocal HCC [7–9]. Fourthly, the radiomics results may slightly vary between different radiomics or statistical analysis software from feature selection to model evaluation. Hence, the well-recognized LASSO algorithm of R software [13, 40], Pyradiomics [40–42], and Scikit-learn [43, 44] packages of Python software were also employed to this paper, for facilitating the future study to verify the robustness of our findings. Finally, HCC has a strong male preponderance [45], and thus, the sex ratio imbalance—the inherent selection bias—cannot be completely avoided in this study.

In summary, mainly based upon multi-parametric radiomics in VOI_tumor + 10mm + liver_ of Gd-EOB-DTPA MRI, the nomogram using random forest is a potential biomarker for preoperatively predicting MVI and RFS in patients with solitary HCC ≤ 5 cm.

## Supplementary Information

ESM 1 (DOCX 1639 kb)

## Abbreviations

AFP: Alpha-fetoprotein
AKP: Alkaline phosphatase
ALT: Alanine aminotransferase
AP: Arterial phase
AUC: Area under the curve
HBP: Hepatobiliary phase
HCC: Hepatocellular carcinoma
LASSO: Least Absolute Shrinkage and Selection Operator
LR: Logistic regression
MVI: Microvascular invasion
NRI: Net reclassification index
OR: Odds ratio
PVP: Portal venous phase
RF: Random forest
TBIL: Total bilirubin
TP: Transitional phase
VOI: Volumetric interest
