# Supplementary material for: Multi-scale and multi-parametric radiomics of gadoxetate disodium–enhanced MRI predicts microvascular invasion and outcome in patients with solitary hepatocellular carcinoma ≤ 5 cm
Source: Eur Radiol. 2021 Jan 14;31(7):4824–38. doi: 10.1007/s00330-020-07601-2 (PMC8213553; doi:10.1007/s00330-020-07601-2)

**Supplementary Materials and Methods**

**Magnetic Resonance Imaging**

Pre-contrast and hepatobiliary phase (HBP) T1 maps were automatically reconstructed by Syngo MapIt software (Version VD13; Siemens Healthcare) from the three-dimensional gradient-echo volumetric-interpolated breath-hold examination with a dual flip-angle of 2° and 12° before and 20 minutes after the injection of gadoxetic acid. Detailed parameters are shown in Table S1.

The paraphrases of MRI morphologic features are as follows: (a) peritumoral enhancement on arterial phase images, defined as an arterial enhancement region adjacent to tumor boundary, which became isointense with the background liver parenchyma in the delayed phase [1]; (b) peritumoral hypointensity on hepatobiliary phase images, presented as an irregular, wedge-shaped, or flame-like hypointense area of liver parenchyma located outside of the tumor margin [1]; (c) capsule enhancement, identified as a thin, linear and enhanced structure surrounded the tumor on the delayed phase images [2]; (d) the longest diameter of tumor the longest axis diameter measured on HBP images; (e) typical MRI pattern (hypervascularity in arterial phase images and washout in portal venous phase images) on the basis of the American Association for the Study of Liver Diseases (AASLD) [3; 4].

**Radiomics Analysis**

Feature Extraction

Using the Pyradiomics packages, a set of 854 radiomics features were extracted from the original images, as well as from 8 banks (LLL, LLH, etc.,) of three-dimensional wavelet filters images [5] which applied either a high (H) or low (H) pass filter in each of the three dimensions, enhancing the slowly or the quickly changing image signal along each dimension, respectively.

The extracted features (Table S2) included tumor shape (e.g, compactness, sphericity), size (e.g, volume, surface area), image intensity (e.g, range and percentile of voxel intensity), and texture (gray level co-occurrence matrix [6], gray-level run-length matrix [7] gray-level size-zone matrix [6], neighborhood graytone difference matrix [8], and gray level dependence matrix [10]). Please note that the shape and size features are only extracted from the tumor region VOI_tumor_ because other regions are either irrelevant such as the liver background blocks VOI_liver_ which were randomly picked by the radiologists for contrast analysis, or derived from the tumor region like VOI_tumor+5mm_ and VOI_50%_, therefore the shape and size feature were redundant with corresponding ones extracted from VOI_tumor_. Only texture and intensity features were extracted from all VOIs and used in the experiments.

Feature Selection

To remove redundant low MVI-correlated features, the interobserver agreement — the intra-class correlation coefficient (ICC, Table S3) was first tested to determine the feature stability. For the 100 randomly selected patients from overall dataset, radiomics features extracted from different delineators with an ICC lower than 0.8 were excluded from the following radiomics analysis (Fig.S4). Subsequently, Univariate Feature Selection (UFS) with statistical F-test method and Least Absolute Shrinkage and Selection Operator (LASSO) [9; 10] were sequentially employed to filter the features which were normalized using Z-score method. Akaike information criteria and cross validation were used to select the regularization parameter alpha (α) of the LASSO estimator.

The feature selection was first applied to the extracted features from each VOI in single sequences except for VOI_liver_ because this region was intended to be used in conjunction with the tumor region for contrast rather than to be used separately. The same intensity and texture features selected from VOI_tumor_ were applied to VOI_liver_ in all subsequent experiments. All selected features from each VOI of every sequence were finally joined and filtered by the same feature selection approach again to train the multi-VOI, multi-sequence model.

**Statistical analyses**

Continuous variables were expressed as mean ± SD. Interclass consistency of predictive variables between training and validation cohorts was tested by the independent samples t' test for continuous variables, and by the Chi-square test or the Fisher’s exact test (two tailed) for categorical variables. In univariate LR analysis, variables (*P* <0.1) in training cohort were entered into multivariate LR analysis using backward (Wald) stepwise selection and RF classifier, respectively. The interobserver variability of imaging traits was evaluated by McNemar’s test. The interobserver agreement of manually delineated VOI_tumor_, as well as imaging traits, between radiologists was evaluated by Cohen kappa coefficient (κ statistics <0.40, poor; 0.40–0.59, fair; 0.60–0.74, good; and 0.75–1.00, excellent). The calibration curve and decision curve analyses were applied to explore the predictive consistency between nomogram and the actual MVI status, and evaluate the clinical net benefits of nomogram, respectively. Survival curves were plotted by Kaplan-Meier method and compared by a 2-sided log-rank test. Univariate and multivariate Cox’s proportional hazards regression determined the predictors of recurrence. Nomogram with Harrell’s C-index estimated the survival prediction model. A two-tailed *P* <0.05 was considered statistically significant.

**Results**

In univariate LR analysis of clinical model, the *P* values of alpha-fetoprotein, total bilirubin, Barcelona Clinic Liver Cancer (BCLC) stage and des-gamma-carboxy prothrombin were less than 0.1. Besides, α-L-fucosidase has been reported to be an important diagnostic and prognostic predictor for HCC [11-13]. Meanwhile, there are significant interclass differences (P<0.05) of fibrinogen, total protein and activated partial thromboplastin time between the training and validation cohort. Therefore, the eight indicators mentioned above (Table 1) were enrolled in multivariate LR analysis.

In imaging model and image segmentation, typical MRI pattern, peritumoral enhancement, capsule enhancement, peritumoral hypointensity and the manually delineated VOIs between radiologists were highly consistent among radiologists (Kappa values: 0.819, 0.948, 0.901, 0.901 and 0.950, all *P* values <0.001), indicating the excellent interobserver agreement.

**References**

1 Hu HT, Shen SL, Wang Z et al (2018) Peritumoral tissue on preoperative imaging reveals microvascular invasion in hepatocellular carcinoma: a systematic review and meta-analysis. Abdom Radiol (NY) 43:3324-3330

2 Wei Y, Huang Z, Tang H et al (2019) IVIM improves preoperative assessment of microvascular invasion in HCC. Eur Radiol 29:5403-5414

3 Heimbach JK, Kulik LM, Finn RS et al (2018) AASLD guidelines for the treatment of hepatocellular carcinoma. Hepatology 67:358-380

4 Forner A, Reig M, Bruix J (2018) Hepatocellular carcinoma. Lancet 391:1301-1314

5 Aerts HJ, Velazquez ER, Leijenaar RT et al (2014) Decoding tumour phenotype by noninvasive imaging using a quantitative radiomics approach. Nat Commun 5:4006

6 Thibault G, Fertil B, Navarro C et al (2013) Shape and texture indexes application to cell nuclei classification. International Journal of Pattern Recognition & Artificial Intelligence 27:1357002

7 Galloway MM (1974) Texture analysis using grey level run lengths. Nasa Sti/recon Technical Report N:75

8 Amadasun M, King R (1989) Textural features corresponding to textural properties. IEEE Transactions on Systems, Man, and Cybernetics 19:1264-1274

9 Tibshirani R (1997) The lasso method for variable selection in the Cox model. Statistics in medicine 16:385-395

10 Sauerbrei W, Royston P, Binder H (2007) Selection of important variables and determination of functional form for continuous predictors in multivariable model building Statistics in medicine 26:5512-5528.

11 Xing H, Qiu H, Ding X et al (2019) Clinical performance of alpha-L-fucosidase for early detection of hepatocellular carcinoma. Biomark Med 13:545-555

12 Mintz K, Waidely E, Zhou Y et al (2018) Carbon dots and gold nanoparticles based immunoassay for detection of alpha-L-fucosidase. Anal Chim Acta 1041:114-121

13 Wang K, Guo W, Li N et al (2014) Alpha-1-fucosidase as a prognostic indicator for hepatocellular carcinoma following hepatectomy: a large-scale, long-term study. Br J Cancer 110:1811-1819

**Supplementary Tables**

**Table S1. Parameters of MRI sequences..**

| Parameters | TR | TE | BW | FOV | Matrix | Section thickness(mm) | Acquistion  time (sec) |
| --- | --- | --- | --- | --- | --- | --- | --- |
| Respiratory-triggered T2-weighted imaging | 4918 | 106 | 195 | 285x380 | 384x273 | 5.5 | Respiration-  dependent |
| Free-breathing DWI ^a^ | 5100 | 55 | 1565 | 285x380 | 192x154 | 5.5 | 74 |
| Breath-hold T1-weighted in-phase and opposed-phase imaging | 6.88 | 2.39/4.77 | 435 | 356x380 | 320x240 | 3.5 | 18 |
| Breath-hold T1-weighted VIBE imaging | 3.47 | 1.36 | 400 | 308x380 | 320x240 | 3 | 15 |
| Breath-hold T1 maps ^b^ | 4.38 | 1.93 | 405 | 285x380 | 216x288 | 3 | 15 |

1. Abbreviation: TR, Repetition time; TE, Echo time; BW, Bandwidth; FOV, Field of view; DWI, Diffusion-weighted Imaging; VIBE, Volumetric interpolated breath-hold examination;
2. DWI ^a^, performed with b values of 0 and 500 sec/mm2;
3. T1 maps ^b^, derived from Syngo MapIt software (Version VD13; Siemens Healthcare, Erlangen, Germany) after data acquisition from a 3D-T1WI-VIBE with a dual flip-angle of 2° and 12°.

**Table S2. The extracted radiomics features in this study.**

| Non-texture Features(n=32) | |
| --- | --- |
| Feature category | Features |
| Shape and Size [1] (n=13) | Volume; Surface Area; Surface Area to Volume ratio Sphericity; Spherical Disproportion; Compactness 1; Compactness 2; Maximum 3D diameter; Major Axis Length; Minor Axis Length; Least Axis Length; Elongation; Flatness |
| First Order Statistic [1] (n=19) | Energy; Total EnergyEntropy; Minimum; 10th percentile; 90th percentile; Maximum; Mean; Median; Interquartile Range; Range; Mean Absolute Deviation; Robust Mean Absolute Deviation; Root Mean Squared; Standard Deviation; Skewness; Kurtosis; Variance; Uniformity |
| Textural Features(n=75) | |
| Gray Level Co-occurrence Matrix [2]  (GLCM, n=24) | Autocorrelation; Joint AverageCluster Prominence; Cluster Shade; Cluster Tendency; Contrast; Correlation; Difference Average; Difference Entropy; Difference Variance; Joint Energy; Joint Entropy; Informational Measure of Correlation 1; Informational Measure of Correlation 2; Inverse Difference Moment; Maximal Correlation Coefficient; Inverse Difference Moment Normalized;Inverse Difference; Inverse Difference Normalized; Inverse Variance; Maximum Probability; Sum Average; Sum Entropy; Sum of Squares |
| Gray Level Run Length Matrix Features [3-7]  (GLRLM, n=16) | Short Run Emphasis; Long Run Emphasis; Gray Level Non-Uniformity;Gray Level Non-Uniformity Normalized; Run Length Non-Uniformity; Run Length Non-Uniformity Normalized; Run Percentage; Gray Level Variance; Run Variance; Run Entropy; Low Gray Level Run Emphasis; High Gray Level Run Emphasis; Short Run Low Gray Level Emphasis ; Short Run High Gray Level Emphasis; Long Run Low Gray Level Emphasis; Long Run High Gray Level Emphasis |
| Gray Level Size Zone Matrix [8] (GLSZM, n=16) | Small Area Emphasis; Large Area Emphasis; Gray Level Non-Uniformity; Gray Level Non-Uniformity Normalized; Size-Zone Non-Uniformity; Size-Zone Non-Uniformity Normalized; Zone Percentage; Gray Level Variance; Zone Variance; Zone Entropy; Low Gray Level Zone Emphasis; High Gray Level Zone Emphasis; Small Area Low Gray Level Emphasis; Small Area High Gray Level Emphasis; Large Area Low Gray Level Emphasis; Large Area High Gray Level Emphasis |
| Neighboring Gray Tone Difference Matrix [9] (NGTDM, n=5) | Coarseness; Contrast;  Busyness; Complexity;  Strength |
| Gray Level Dependence Matrix [10] (GLDM, n=14) | Small Dependence Emphasis; Large Dependence Emphasis; Gray Level Non-Uniformity; Dependence Non-Uniformity; Dependence Non-Uniformity Normalized; Gray Level Variance; Dependence Variance; Dependence Entropy; Low Gray Level Emphasis; High Gray Level Emphasis; Small Dependence Low Gray Level Emphasis; Small Dependence High Gray Level Emphasis; Large Dependence Low Gray Level Emphasis; Large Dependence High Gray Level Emphasis |

**Table S3. The number of the selected features in each volumetric interest and single sequence.**

| MR sequence | Feature number(N) | | |
| --- | --- | --- | --- |
|  | ICC selection | UFS selection | LASSO selection |
|  | VOI_tumor_/VOI_50%_/VOI_5mm_/VOI_10mm_ | VOI_tumor_/VOI_50%_/VOI_5mm_/VOI_10mm_ | VOI_tumor_/VOI_50%_/VOI_5mm_/VOI_10mm_ |
| T2WI | 829/796/822/829 | 30/30/30/70 | 3/7/2/3 |
| DWI | 734/635/781/772 | 100/80/30/80 | 6/4/3/5 |
| Pre | 832/797/820/816 | 60/100/70/70 | 10/7/9/5 |
| Pre-T1 maps | 277/621/823/837 | 30/50/30/50 | 10/8/1/5 |
| AP | 845/780/811/824 | 30/50/110/110 | 13/7/7/7 |
| PVP | 818/779/822/823 | 40/30/100/70 | 7/6/6/4 |
| TP | 823/767/809/809 | 40/30/110/80 | 8/5/4/4 |
| HBP | 763/719/770/777 | 30/30/110/80 | 9/2/5/3 |
| HBP-T1 maps | 246/763/809/818 | 30/110/100/40 | 5/13/4/3 |

1. ICC, intraclass correlation coefficient; UFS, Univariate Feature Selection; LASSO, Least Absolute Shrinkage and Selection Operator; T2WI, T2-weighted image; DWI, dffusion-weighted image; Pre, pre-contrast T1-weighted image; Pre-T1 map, pre-contrast T1 maps; AP, arterial phase; PVP, portal venous phase; TP, transitional phase; HBP, hepatobiliary phase; HBP-T1 maps, hepatobiliary phase T1 map.

2.VOI, volumetric interest; VOI_tumor_, the entire volume of tumor; VOI_50%_, the 50% central region of VOI_tumor_; VOI_5mm_ or VOI_10mm_, the peritumoural area within the distance of 5mm or 10mm from the tumor margin, respectively.

3. Final fusion radiomics signatures: the best multi-parametric combination (arterial phase, portal venous phase, hepatobiliary phase T1-weighted images and diffusion-weighted imaging) based on VOI_tumor+10mm+liver_ (VOI_tumor_, VOI_10mm_ and some normal liver zones).

**Table S4. The performance of VOI_tumor+10mm+liver_ using random forest in each single sequence for predicting histologic MVI.**

| Sequence | Training dataset (n=250) | | | | Validation dataset (n=106) | | | |
| --- | --- | --- | --- | --- | --- | --- | --- | --- |
|  | Sen | Spe | AUC(95%CI) | *P ^a^* | Sen | Spe | AUC(95%CI) | *P ^a^* |
| T2WI | 0.966 | 0.886 | 0.975(0.955-0.995) | <0.001 | 0.633 | 0.776 | 0.755(0.677-0.833) | <0.001 |
| DWI | 0.932 | 0.930 | 0.978(0.958-0.998) | <0.001 | 0.759 | 0.743 | **0.812**(0.734-0.890) | <0.001 |
| PRE | 0.860 | 0.848 | 0.912(0.873-0.951) | <0.001 | 0.900 | 0.605 | 0.810(0.732-0.888) | <0.001 |
| PRE-T1 maps | 0.732 | 0.650 | 0.752(0.693-0.811) | <0.001 | 0.833 | 0.581 | 0.766(0.688-0.844) | <0.001 |
| AP | 0.814 | 0.919 | 0.944(0.924-0.964) | <0.001 | 0.862 | 0.733 | **0.830**(0.752-0.908) | <0.001 |
| PVP | 0.800 | 0.893 | 0.912(0.873-0.951) | <0.001 | 0.800 | 0.763 | **0.837**(0.759-0.915) | <0.001 |
| TP | 0.638 | 0.813 | 0.802(0.734-0.861) | <0.001 | 0.767 | 0.671 | 0.792(0.714-0.870) | <0.001 |
| HBP | 0.817 | 0.804 | 0.885(0.846-0.924) | <0.001 | 0.966 | 0.68 | **0.855**(0.791-0.919) | <0.001 |
| HBP-T1 maps | 0.667 | 0.781 | 0.807(0.768-0.846) | <0.001 | 0.656 | 0.8 | 0.731(0.653-0.809) | <0.001 |

VOI_tumor+10mm+liver_ : the radiomics feature fusion on the basis of the entire tumor, peritumoral area within 10mm and some normal liver zones;

Abbreviation: AUC, area under curve; *P ^a^*: the *P* value of AUC; Sen: sensitivity; Spe: specificity; T2WI, T2-weighted image; DWI, diffusion-weighted image; PRE: pre-contrast T1-weighted image (T1WI); AP: arterial phase; PVP, portal venous phase; TP: transitional phase; HBP, hepatobiliary phase.

**Bold**: the highest AUCs of the validation cohort in single sequences.

**Table S5. The number and the most MVI-correlated signatures of multi-VOI features selected by LASSO in each sequence and the final radiomics model.**

| Sequence (feature number) ^a^ | | Top Five MVI-correlated Multi-VOI Features Selected by LASSO ^a^ |
| --- | --- | --- |
| T2WI  (n=11) | VOI_tumor__wavelet-LHL_glrlm_LongRunHighGrayLevelEmphasis  VOI_10mm__wavelet-LHL_glszm_GrayLevelNonUniformity; VOI_5mm__original_glszm_GrayLevelNonUniformity  VOI_5mm__wavelet-HLL_glszm_GrayLevelNonUniformity; VOI_tumor__wavelet-LLL_firstorder_Range | |
| DWI  (n=19) | VOI_tumor__original_shape_MajorAxisLength;VOI_10mm__wavelet-LLH_glszm_SizeZoneNonUniformity  VOI_tumor__wavelet-LLH_glszm_GrayLevelNonUniformity; VOI_5mm__wavelet-LLH_glszm_SizeZoneNonUniformity  VOI_5mm__wavelet-LLL_glrlm_GrayLevelNonUniformity | |
| Pre  (n=34) | VOI_5mm__wavelet-HLL_firstorder_Energy; VOI_5mm__wavelet-HHL_firstorder_Energy  VOI_10mm__wavelet-HLL_glszm_GrayLevelNonUniformity;VOI_tumor__wavelet-LHL_glszm_ZoneEntropy  VOI_tumor__wavelet-LHH_glszm_SmallAreaLowGrayLevelEmphasis | |
| Pre-T1 maps (n=16) | VOI_5mm__wavelet-LLL_glszm_GrayLevelNonUniformity; VOI_10mm__wavelet-LLL_glszm_GrayLevelNonUniformity  VOI_10mm__wavelet-HLL_gldm_DependenceNonUniformity;  VOI_tumor__wavelet-HHL_glszm_GrayLevelNonUniformity;  VOI_10mm__wavelet-HHL_glszm_LargeAreaHighGrayLevelEmphasis | |
| AP (n=37) | VOI_tumor__wavelet-HLL_glcm_Autocorrelation; VOI_10mm__wavelet-LLH_glszm_SizeZoneNonUniformity  VOI_5mm__wavelet-LLH_glszm_GrayLevelNonUniformity; VOI_5mm__wavelet-LLH_glszm_SizeZoneNonUniformity  VOI_10mm__wavelet-LLH_glszm_GrayLevelNonUniformity | |
| PVP (n=22) | VOI_5mm__original_glszm_GrayLevelNonUniformity; VOI_10mm__original_glszm_GrayLevelNonUniformity  VOI_5mm__wavelet-LLH_glszm_GrayLevelNonUniformity;VOI_5mm__wavelet-HHL_glszm_SizeZoneNonUniformity  VOI_tumor__wavelet-HLL_gldm_LargeDependenceHighGrayLevelEmphasis | |
| TP (n=23) | VOI_5mm__wavelet-HLL_firstorder_Energy; VOI_tumor__original_glszm_GrayLevelNonUniformity  VOI_tumor__original_glszm_SizeZoneNonUniformity; VOI_10mm__wavelet-HLL_firstorder_Energy  VOI_10mm__wavelet-LLH_glszm_GrayLevelNonUniformity | |
| HBP (n=23) | VOI_5mm__wavelet-HLL_glszm_GrayLevelNonUniformity; VOI_tumor__original_shape_Sphericity  VOI_5mm__wavelet-HHL_firstorder_Energy; VOI_tumor__wavelet-HLL_glszm_SizeZoneNonUniformity  VOI_tumor__original_glszm_GrayLevelNonUniformity | |
| HBP-T1 maps (n=17) | VOI_tumor__wavelet-LHH_glrlm_RunVariance; VOI_tumor__original_glrlm_LongRunHighGrayLevelEmphasis  VOI_tumor__wavelet-LLL_glszm_SizeZoneNonUniformity; VOI_tumor__wavelet-LHH_glrlm_ShortRunEmphasis  VOI_tumor__wavelet-LLL_firstorder_Energy | |
| Final radiomics (n=74) | HBP_VOI_5mm__wavelet-HLL_glszm_GrayLevelNonUniformity; HBP_VOI_5mm__wavelet-HHL_firstorder_Energy  DWI_VOI_tumor__original_shape_MajorAxisLength; HBP_VOI_tumor__original_shape_Sphericity  HBP_VOI_tumor__wavelet-HLL_glszm_SizeZoneNonUniformity | |

^a^: Multi-VOI radiomics features selected by least absolute shrinkage and selection operator (LASSO) with five-fold cross-validation. T2WI, T2-weighted image; DWI, dffusion-weighted image; Pre, pre-contrast T1-weighted image; Pre-T1 map, pre-contrast T1 maps; AP, arterial phase; PVP, portal venous phase; TP, transitional phase; HBP, hepatobiliary phase; HBP-T1 maps, hepatobiliary phase T1 map. Final fused radiomics model: the combination of PVP, HBP, AP and DWI in VOI _tumor+10mm+liver_

**Table S6. Results of multi-parametric fusion based on VOI _tumor+10mm+liver_ for predicting histologic MVI.**

| Methods and  Cohorts | | AUC | | | | | | | | | | |
| --- | --- | --- | --- | --- | --- | --- | --- | --- | --- | --- | --- | --- |
|  |  | HBP | PVP | PVP  +HBP | PVP  +AP | PVP  +HBP  +AP | PVP  +AP  +Pre_T1 | **PVP**  **+HBP**  **+AP +DWI** | PVP  +HBP  +AP  +T2WI | PVP  +HBP  +AP  +Pre_T1 | PVP+AP  +HBP  +DWI  +HBP_T1 | PVP+HBP  +AP+DWI  +T2WI+TP  +Pre_T1 |
| RF | VD | 0.855 | 0.837 | 0.883 | 0.874 | 0.901 | 0.860 | **0.918** | 0.903 | 0.886 | 0.883 | 0.889 |
|  | TD | 0.885 | 0.912 | 0.960 | 0.996 | 1.0 | 0.993 | 0.999 | 0.996 | 0.988 | 0.978 | 0.981 |
| LR | VD | 0.805 | 0.806 | 0.794 | 0.810 | 0.807 | 0.807 | **0.809** | 0.807 | 0.806 | 0.778 | 0.796 |
|  | TD | 0.715 | 0.727 | 0.804 | 0.819 | 0.764 | 0.832 | 0.773 | 0.853 | 0.862 | 0.801 | 0.853 |

Abbreviation: RF, random forest; LR, logistic regression; VD, validation dataset; TD, training dataset; T2WI, T2-weighted image; DWI, diffusion-weighted image; Pre, pre-contrast T1-weighted image (T1WI); Pre_T1, pre-contrast T1 maps; AP, arterial phase T1WI; PVP, portal venous phase T1WI; TP, transitional phase T1WI; HBP, hepatobiliary phase T1WI; HBP_T1, hepatobiliary phase T1 maps.

**Table S7. Results of multi-parametric fusion based on VOI_tumor_ in cohorts for predicting histologic MVI.**

| Methods and  Cohorts | | AUC | | | | | | | | | | |
| --- | --- | --- | --- | --- | --- | --- | --- | --- | --- | --- | --- | --- |
|  |  | HBP | PVP | PVP  +  HBP | PVP  +  AP | PVP  +HBP  +AP | PVP  +HBP  +Pre | PVP  +AP  +Pre_T1 | **PVP**  **+HBP**  **+AP**  **+Pre_T1** | PVP  +AP  +Pre_T1  +HBP_T1 | PVP+AP  +HBP  +Pre_T1  +T2WI | PVP+HBP  +AP+Pre_T1  +T2WI+TP  +HBP_T1 |
| RF | VD | 0.799 | 0.810 | 0.832 | 0.840 | 0.840 | 0.831 | 0.863 | **0.871** | 0.857 | 0.862 | 0.861 |
|  | TD | 0.991 | 0.996 | 0.946 | 0.944 | 0.944 | 0.911 | 0.987 | 1.000 | 0.990 | 0.995 | 0.999 |
| LR | VD | 0.746 | 0.768 | 0.766 | 0.770 | 0.770 | 0.784 | 0.785 | **0.792** | 0.784 | 0.798 | 0.768 |
|  | TD | 0.744 | 0.755 | 0.774 | 0.777 | 0.777 | 0.780 | 0.787 | 0.813 | 0.786 | 0.814 | 0.778 |

Abbreviation: RF, random forest; LR, logistic regression; VD, validation dataset; TD, training dataset; T2WI, T2-weighted image; DWI, diffusion-weighted image; Pre, pre-contrast T1-weighted image (T1WI); Pre_T1, pre-contrast T1 maps; AP, arterial phase T1WI; PVP, portal venous phase T1WI; TP, transitional phase T1WI; HBP, hepatobiliary phase T1WI; HBP_T1, hepatobiliary phase T1 maps.

**Table S8. The top six most discriminating signatures in the final radiomics model for predicting MVI.**

| Feature name | Formula | Content |
| --- | --- | --- |
| HBP_VOI_5mm__wavelet_  HLL_glszm_GrayLevelNonUniformity | $GLN=\frac{\sum_{i=1}^{N_{g}} \left( \sum_{j=1}^{N_{s}} P\left( i,j \right) \right)^{2}}{N_{z}}$ | GLN measures the variability of gray-level intensity values in the VOI_5mm_ area of the HBP image transformed by wavelet filter HLL, with a lower value indicating more homogeneity in intensity values. |
| HBP_VOI_5mm__wavelet_  HHL_firstorder_Energy | $Energy=\sum_{i=1}^{N_{p}} \left( X\left( i \right)+c \right)^{2}$ | Energy is a measure of the magnitude of voxel values in the VOI_5mm_ area of the HBP image transformed by wavelet filter HHL. A larger values implies a greater sum of the squares of these values. |
| HBP_VOI_tumor__original_  shape_Sphericity | $Spherical disproportion=\frac{A}{4\pi R^{2}}=\frac{A}{\sqrt[3]{36\pi V^{2}}}$ | Spherical Disproportion is the ratio of the surface area of the VOI_tumor_ region to the surface area of a sphere with the same volume as the VOI_tumor_ region in the HBP image, and by definition, the inverse of Sphericity. Therefore, the value range is spherical disproportion ≥ 1, with a value of 1 indicating a perfect sphere. |
| HBP_VOI_tumor__original_  glszm_GrayLevelNonUniformity | $GLN=\frac{\sum_{i=1}^{N_{g}} \left( \sum_{j=1}^{N_{s}} P\left( i,j \right) \right)^{2}}{N_{z}}$ | GLN measures the variability of gray-level intensity values in the VOI_tumor_ area of HBP images, with a lower value indicating more homogeneity in intensity values. |
| HBP_VOI_tumor__wavelet_  HLL_glszm_SizeZoneNonUniformity | $SZN=\frac{\sum_{j=1}^{N_{s}} \left( \sum_{i=1}^{N_{g}} P\left( i,j \right) \right)^{2}}{N_{z}}$ | SZN measures the variability of size zone volumes in the VOI_tumor_ area of HBP images transformed by wavelet filter HLL, with a lower value indicating more homogeneity in size zone volumes. |
| DWI_VOI_tumor__original_  shape_MajorAxisLength | $Major axis=4\sqrt{\lambda_{major}}$ | MAL yield the largest axis length of the VOI_tumor_ area in DWI image and is calculated using the largest principal component . |

Where:

**X** be the intensity value of original image, **XLLL**, **XLLH**, **XLHL**, **XLHH**, **XHLL**, **XHLH**，**XHHL**，**XHHH** be the intensity value of the transformation images from original image by 8 three dimensional wavelet filters. **L** represent low-pass filter, **H** represent low-pass filter. For example, **XLHL** represent the intensity value resulting from directional filtering of X with a low-pass filter along x-direction, a high pass filter along y-direction and a low-pass filter along z-direction

$\boldsymbol{c}$is optional value, defined by voxelArrayShift , which shifts the intensities to prevent negative values in $\mathbf{X}$.

$\boldsymbol{R}$ is the radius of a sphere with the same volume as the tumor, and equal to $\sqrt[3]{\frac{3V}{4\pi}}$ .

$\boldsymbol{V}$ the volume of the mesh in ${mm}^{3}$.

$\boldsymbol{N}_{\boldsymbol{g}}$ is the number of discrete intensity values in the image.

$\boldsymbol{N}_{\boldsymbol{z}}$ is the size of the largest homogeneous region.

$\boldsymbol{N}_{\boldsymbol{s}}$ is the number of discreet zone sizes in the image.

$\boldsymbol{P}\left( \boldsymbol{i,j} \right)$ is be the size zone matrix.

$\boldsymbol{\lambda}_{\boldsymbol{major}}$ is the largest axis length of the largest principal component.

**References**

1 Lorensen WE, Cline HE (1987) Marching Cubes: A High Resolution 3D Surface Construction AlgorithmProceedings of the 14th Annual Conference on Computer Graphics and Interactive Techniques.  ACM SIGGRAPH Computer Graphics New York, NY, USA, pp 163--169

2 Haralick RM, Shanmugam K, Dinstein IH (1973) Textural Features for Image Classification. IEEE Transactions on Systems, Man, and Cybernetics SMC-3:610-621

3 Tustison N, Gee J (2008) Run-Length Matrices For Texture Analysis. OR Insight:January - June

4 Xu D, Kurani AS, Furst JD, Raicu DS (2004) Run-length encoding for volumetric texture. The 4th IASTED International Conference on Visualization, Imaging, and Image Processing

5 Tang X (1998) Texture information in run-length matrices. IEEE Transactions on Image Processing 7:1602-1609

6 A.Chu, Sehgal CM, Greenleaf JF (1990) Use of gray value distribution of run lengths for texture analysis. Pattern Recognition Letters 11:415-419

7 Galloway MM (1975) Texture analysis using gray level run lengths. Computer Graphics and Image Processing 4:172-179

8 Thibault G, Fertil B, Navarro C, Pereira S, Mari JL (2009) Texture Indexes and Gray Level Size Zone Matrix Application to Cell Nuclei Classification10th International Conference on Pattern Recognition and Information Processing,

9 Amadasun M, King R (1989) Textural features corresponding to textural properties. IEEE Transactions on Systems, Man, and Cybernetics 19:1264-1274

10 Sun C, Wee WG (1983) Neighboring gray level dependence matrix for texture classification. Computer Vision, Graphics, and Image Processing 23:341-352

**Supplementary Figure Legends**

**Fig.S1. Calibration and decision curve analyses of MVI nomograms.**The calibration curve analysis evaluated the coincidence between the actual MVI status and MVI nomograms using random forest (RF) in (a) training and (b) validation datasets, and using logistic regression (LR) in (c) training and (d) validation datasets. The decision curves of the clinical model, imaging model, final radiomics model and MVI nomogram were plotted in (e) training and (f) validation datasets using RF, and were presented in (g) training and (h) validation datasets using LR, respectively.


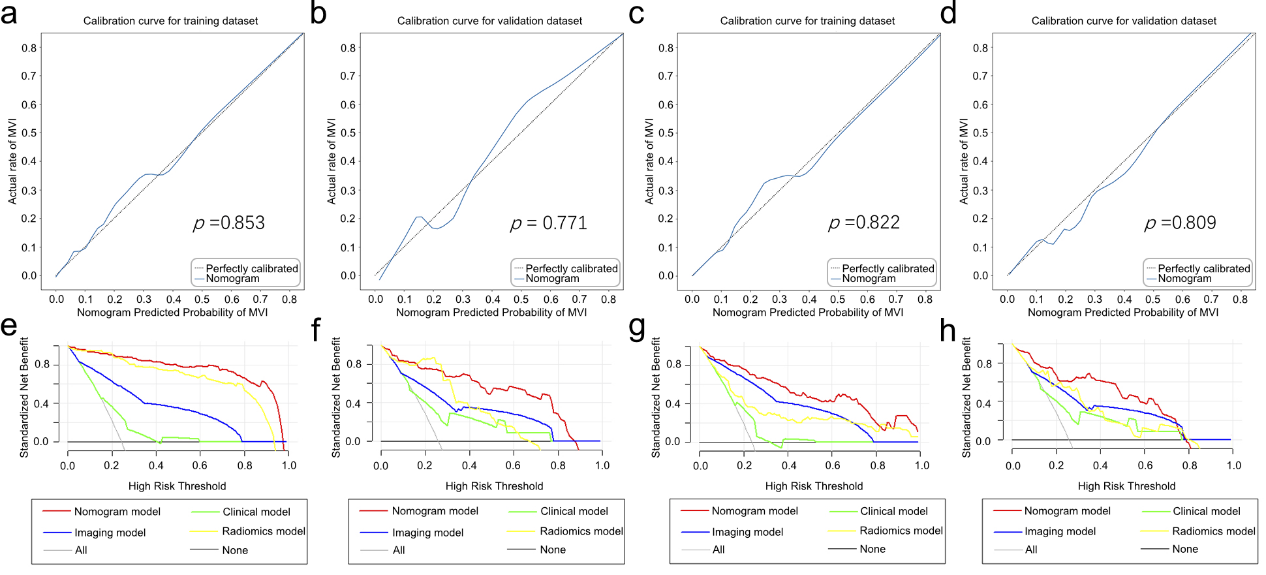


**Fig.S2. Kaplan-Meier curves of recurrence-free survival according to the logistic regression.**According to the MVI nomogram using logistic regression (MVI-LR) and the Kaplan-Meier analysis, the recurrence-free survival curves were scaled by (a) the predicted MVI-LR status and (b) MVI-LR grades, respectively. Based on the 2-sided log-rank test, neither MVI-LR status nor MVI-LR grades can significantly and correctly distinguish the actual survival classifications.


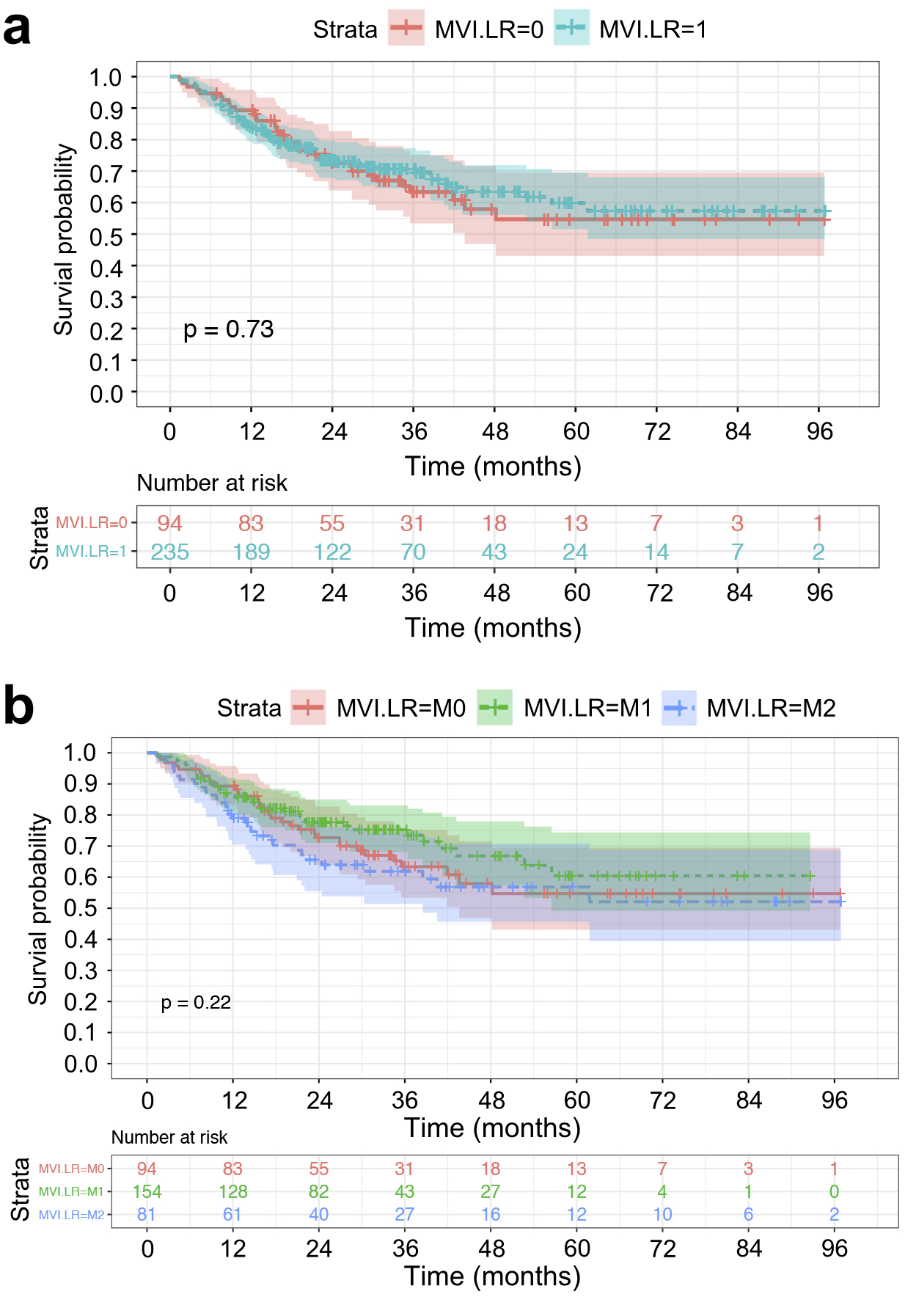


**Fig.S3. Calibration curve analysis of 3-year recurrence-free survival nomograms.**The calibration curve analysis evaluated the coincidence between the nomogram based on the histologic MVI subgroups and the actually 3-year recurrence-free survival (RFS) status in (a) training and (b) validation datasets, and the coincidence between the nomogram based on the predictive MVI-RF subgroups and the actually 3-year RFS status in (c) training and (d) validation datasets, respectively.


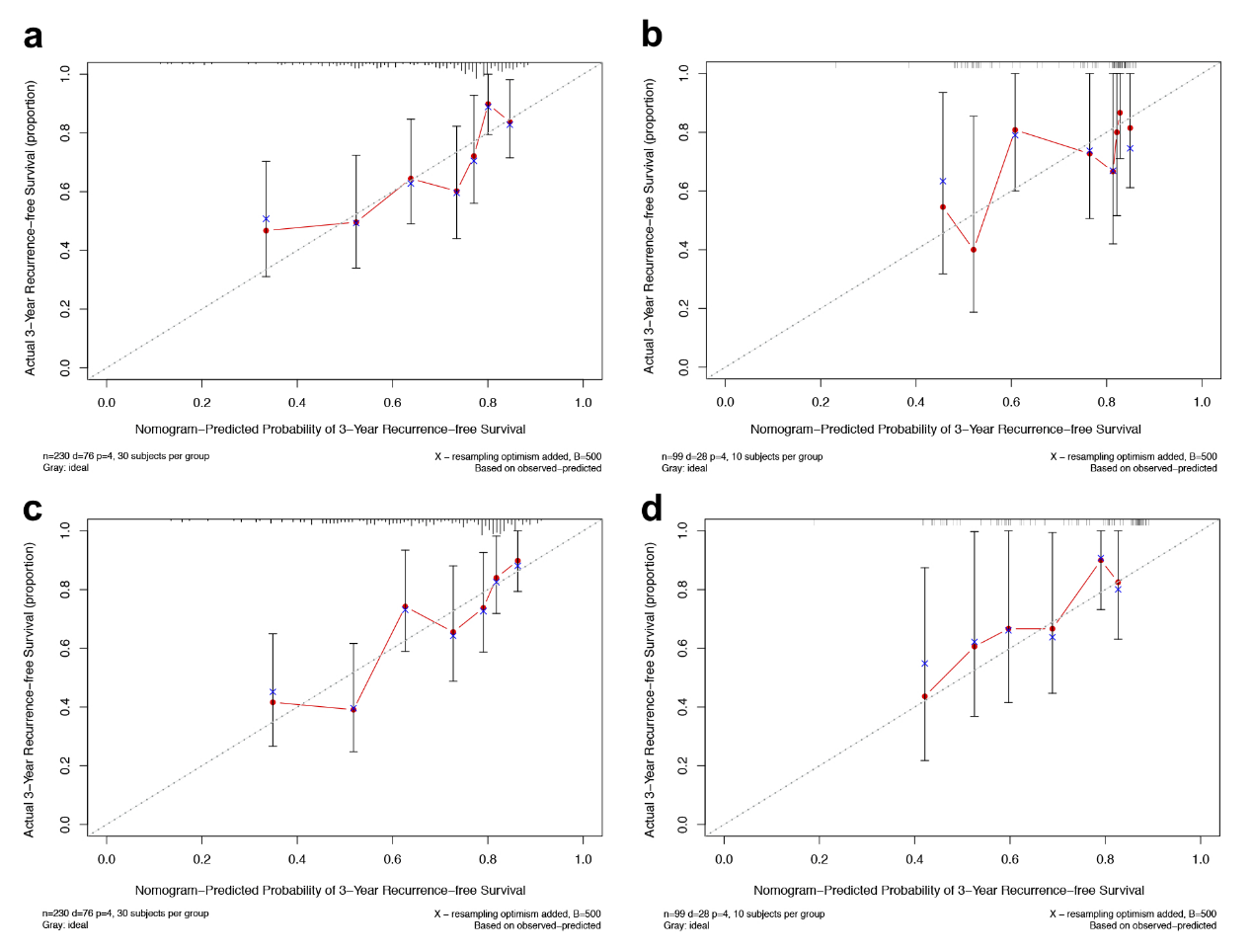


**Fig.S4. Histogram of inter-observer reliability between delineators.**For the 100 randomly selected patients from overall dataset, we extracted radiomic features from diffusion-weighted imaging (DWI), arterial phase (AP), portal venous phase (PVP) and hepatobiliary phase (HBP) images for inter-observer reliability. The intra-class correlation coefficient (ICC) was used to determine the stability of the features. Non-robust: features numbers with the ICC ≤0.8 were excluded from the subsequent radiomics analysis; Robust: features numbers with the ICC >0.8 were enrolled into the subsequent radiomics analysis.


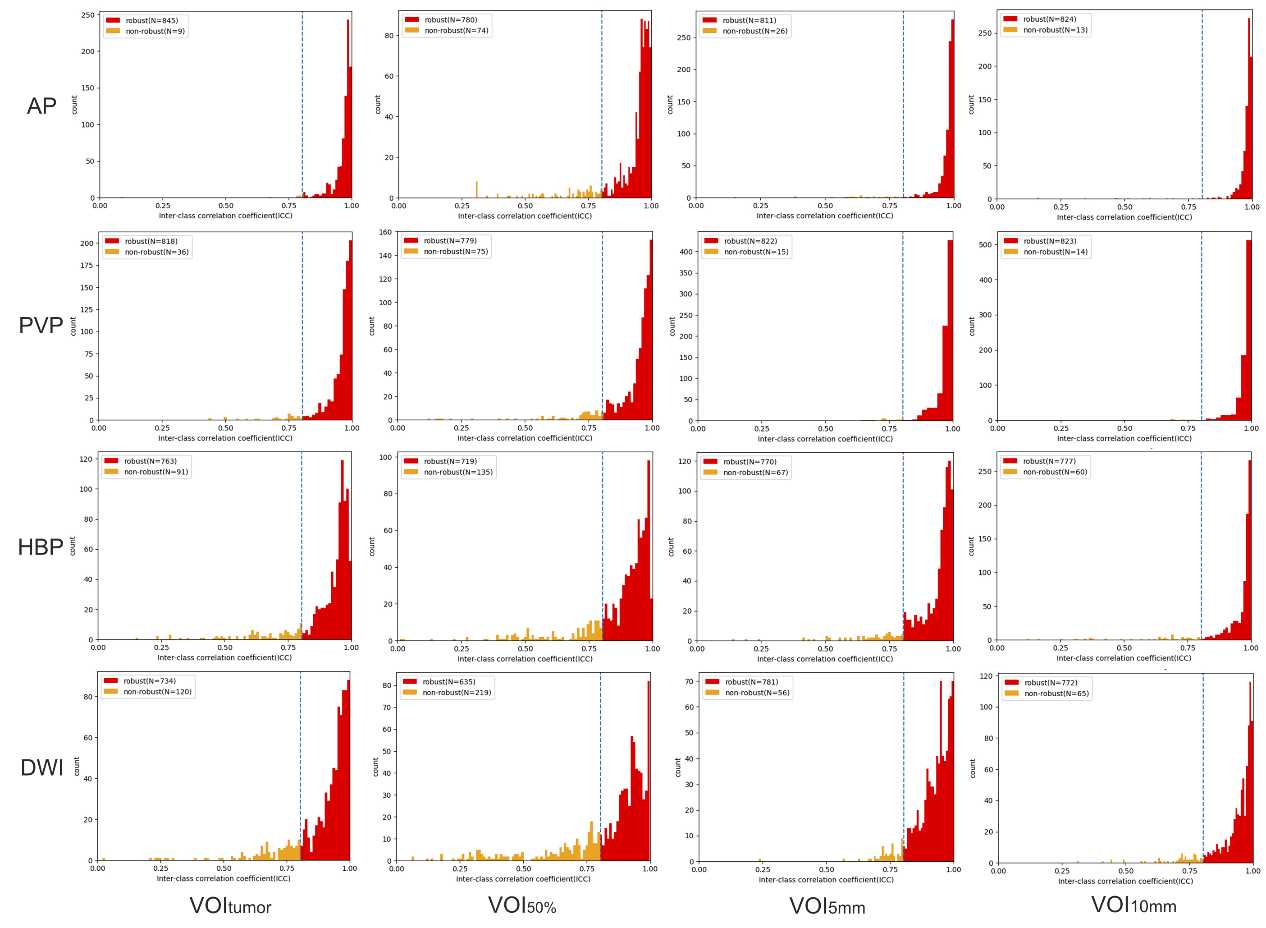

Supplement: Supplementary file 1 — (DOCX 1639 kb) [file 330_2020_7601_MOESM1_ESM.docx]
